# Predicting the Dominant Role of Dense Aggregates in Magnetic Hyperthermia via Intracellular‐Mimetic Nanoparticle Models

**DOI:** 10.1002/smll.202506620

**Published:** 2025-08-29

**Authors:** Pelayo García‐Acevedo, Alba Paz‐Castro, Jorge Estébanez, Yolanda Piñeiro, José Rivas

**Affiliations:** ^1^ NANOMAG Laboratory Applied Physics Department iMATUS Materials Institute and Health Research Institute of Santiago de Compostela (IDIS) Universidade de Santiago de Compostela Santiago de Compostela 15782 Spain; ^2^ Neuroimaging and Biotechnology Laboratory (NOBEL) Clinical Neurosciences Research Laboratory (LINC) Health Research Institute of Santiago de Compostela (IDIS) Santiago de Compostela 15782 Spain; ^3^ Department of Biochemistry and Molecular Biology Faculty of Biology‐Biological Research Centre (CIBUS) Universidade de Santiago de Compostela Santiago de Compostela 15782 Spain

**Keywords:** aggregation effects, magnetic hyperthermia, magnetic interactions, magnetic nanoparticles

## Abstract

Dense aggregation of magnetic nanoparticles (MNPs) in cellular environments is a major contributor to reduced magnetic hyperthermia (MH) efficiency; however, in situ magnetic characterization remains challenging, necessitating reliable extracellular models to predict such behavior. In this study, soft and dense aggregates (≈200 nm) are engineered using 10 nm iron oxide MNPs, encapsulated in polymeric (soft) or inorganic (dense) shells, with morphological features and strong magnetic dipolar interactions confirming their similarity to nanoparticle assemblies found in living cells. Unlike previous studies that induced aggregation by modifying the surrounding medium, the approach enables controlled, invariant aggregation states, allowing systematic evaluation of the transition from soft to dense aggregation under both aqueous and high‐viscosity, cell‐mimicking conditions. Results demonstrate that dense aggregation leads to a >20% reduction in specific absorption rate (SAR), primarily due to decreased remanent magnetization (M_R_), highlighting the critical role of aggregate structure. Viscosity is found to have a non‐negligible effect once MNPs are aggregated, suggesting dominant Néel relaxation modulated by dipolar interactions. A strong SAR–M_R_ correlation is observed, while SAR–coercivity (H_C_) dependence is disrupted by aggregation. These findings offer new insights for optimizing MH efficiency and guiding the design of magnetic nanoactuators for MH therapy.

## Graphical abstract

Intracellular‐mimetic magnetic nanoparticle assemblies reveal how dense aggregation—typical of lysosomal environments—dominates magnetic hyperthermia performance. Using polymeric and inorganic encapsulation to fix aggregate structure, this study shows that dense packing suppresses remanent magnetization and heating efficiency, offering predictive insights for designing more effective magnetic nanoactuators in complex biological media.

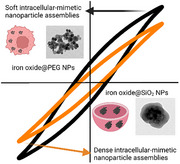

## 1 Introduction

The use of nanotechnology in therapeutic approaches has shown promising results over the past years in the treatment of oncological diseases.^[^ ^1^, ^2^, ^3^, ^4^ ^]^ Magnetic nanoparticles (MNPs), particularly those based on iron oxides, have been extensively investigated as diagnostic systems, being employed as contrast agents in magnetic resonance imaging (MRI)^[^ ^5^, ^6^, ^7^, ^8^, ^9^, ^10^ ^]^ and as therapeutic systems, such as drug delivery vehicles^[^ ^11^, ^12^, ^13^, ^14^, ^15^, ^16^, ^17^ ^]^ and, more interestingly, as thermal actuators in magnetic hyperthermia (MH).^[^ ^18^, ^19^, ^20^, ^21^, ^22^, ^23^, ^24^, ^25^ ^]^ This treatment, whose initial studies date back to the 1950s,^[^ ^26^ ^]^ allows for the thermal ablation of cancer cells and is of particular interest in the treatment of solid tumors. The application of external magnetic fields permits localized heat generation through MNPs, standing out as a treatment with a targeted action area specifically reduced to the tumor zone.^[^ ^27^, ^28^ ^]^ The first clinical MH trials began in the 2000s in patients with glioblastoma, one of the most aggressive and difficult‐to‐treat solid tumors known. In 2010, advances made in the previous decade allowed for the clinically approved implementation of MH in GBM.^[^ ^29^, ^30^ ^]^ However, the clinical translation of this treatment has been notably influenced by the decreased performance of MNPs within living organisms, being one of the major unresolved issues in the field. The complex distribution and aggregation of MNPs in living organisms have proven challenging and are suggested to be key factors in the reduction of SAR.^[^ ^31^ ^]^ In this context, the aggregation of nanoparticles has been extensively studied in recent years, through both theoretical and experimental approaches. Some theoretical models replicate patterns observed in biological tissues and demonstrate that cluster geometry strongly affects heat dissipation: chain‐like structures dissipate ≈50% as much as individual particles, while compact clusters release only one fifth of that.^[^ ^32^ ^]^ Experimental evidence also shows that SAR is strongly influenced by the aggregation state of magnetic nanoparticles (MNPs). For instance, in 14 nm maghemite MNPs, an increase in hydrodynamic diameter (from <80 to >150 nm)—indicative of clustering—leads to a reduction in SAR, attributed to enhanced dipolar interactions within aggregates.^[^ ^33^ ^]^ A similar trend was observed for 11.7 nm MNPs, where dense clustering—modulated by ionic strength and pH—significantly lowered MH efficiency. Although polymer coatings have little impact on heating performance in well‐dispersed systems, aggregation substantially reduces the specific loss power (SLP).^[^ ^34^ ^]^

Furthermore, the inherent polydispersity of MNPs in aqueous media adds complexity, often resulting in a coexistence of aggregated and well‐dispersed particles.^[^ ^35^ ^]^ In such systems, clustering of magnetite nanoparticles with randomly oriented easy axes has been shown to reduce SAR efficiency in bidisperse colloids.^[^ ^36^ ^]^ One of the most influential factors in nanoparticle aggregation has been shown to be dipolar interactions, which play a dual role: small, anisotropic clusters can enhance SAR, while larger, more spherical aggregates reduce it due to decreased shape anisotropy.^[^ ^37^ ^]^ More specifically, the role of intra‐aggregate dipolar interactions (within the aggregate) and inter‐aggregate dipolar interactions (between aggregates) was investigated in MNPs by varying aggregation through concentration and modifying hydrodynamic size using saline buffers. It was demonstrated that increasing inter‐aggregate interactions (through increased concentration) can induce an increase in SAR (increase in M_R_ and M_Max_), while increasing intra‐aggregate interactions (increase in D_H_) led to a decrease in SAR (reduction in M_R_ and M_Max_, along with a variation in H_C_).^[^ ^38^ ^]^ The effects observed under physiological conditions have also been seen in in vitro studies, suggesting that MNP‐cell interactions lead to significant aggregation of MNPs following their cellular internalization.^[^ ^39^, ^40^, ^41^, ^42^ ^]^ It has been proposed that these notable effects appear to be independent of cell type, incubation time, or nanoparticle coating, supporting the idea that contact with cells is sufficient to induce nanoparticle aggregation.^[^ ^43^, ^44^ ^]^ Pioneering studies recognized a significant decline in the magnetic hyperthermia efficiency of nanoparticles in cellular environments, emphasizing the dynamic in situ evaluation in live cells and identifying nanoparticle aggregation as a key factor.^[^ ^31^ ^]^ Subsequent investigations have further highlighted that nanoparticle immobilization within tissues, the inhibition of rotational freedom in the intracellular milieu, and dipolar interactions arising from nanoparticle agglomeration strongly influence magnetic relaxation processes, leading to reduced SAR efficiency. These findings collectively underscore the importance of considering biological environments when evaluating nanoparticle performance and have driven efforts to develop theoretical models and predictive tools to better understand and anticipate heating efficiency under complex intracellular conditions. Although earlier studies have provided valuable insights into how biological environments influence nanoparticle heating efficiency,^[^ ^45^, ^46^, ^47^ ^]^ recent research has revealed further complexities. Specifically, dense aggregation of MNPs within lysosomes has been linked to strong interparticle interactions that reduce magnetic coercivity, underscoring the significant role of the cellular microenvironment on nanoparticle behavior.^[^ ^48^ ^]^ These interparticle interactions, linked to magnetic anisotropy,^[^ ^49^ ^]^ is more pronounced in lysosomal compartments compared to other cellular areas, as the biochemical environment of the lysosomes favors stronger interparticle interactions. Alternatively, recent studies have shown that the application of magnetic fields induces linear alignment into chains, rather than random aggregation, which also influences heat generation.^[^ ^50^, ^51^ ^]^ Although pioneering studies have successfully assessed nanoparticle distribution in tumor environments, emphasizing its role in heat generation,^[^ ^52^, ^53^ ^]^ the complexity and accessibility of current techniques highlight the need for simpler experimental approaches to predict nanoparticle efficiency under dense aggregation conditions in extracellular environments.

**Figure 1:**
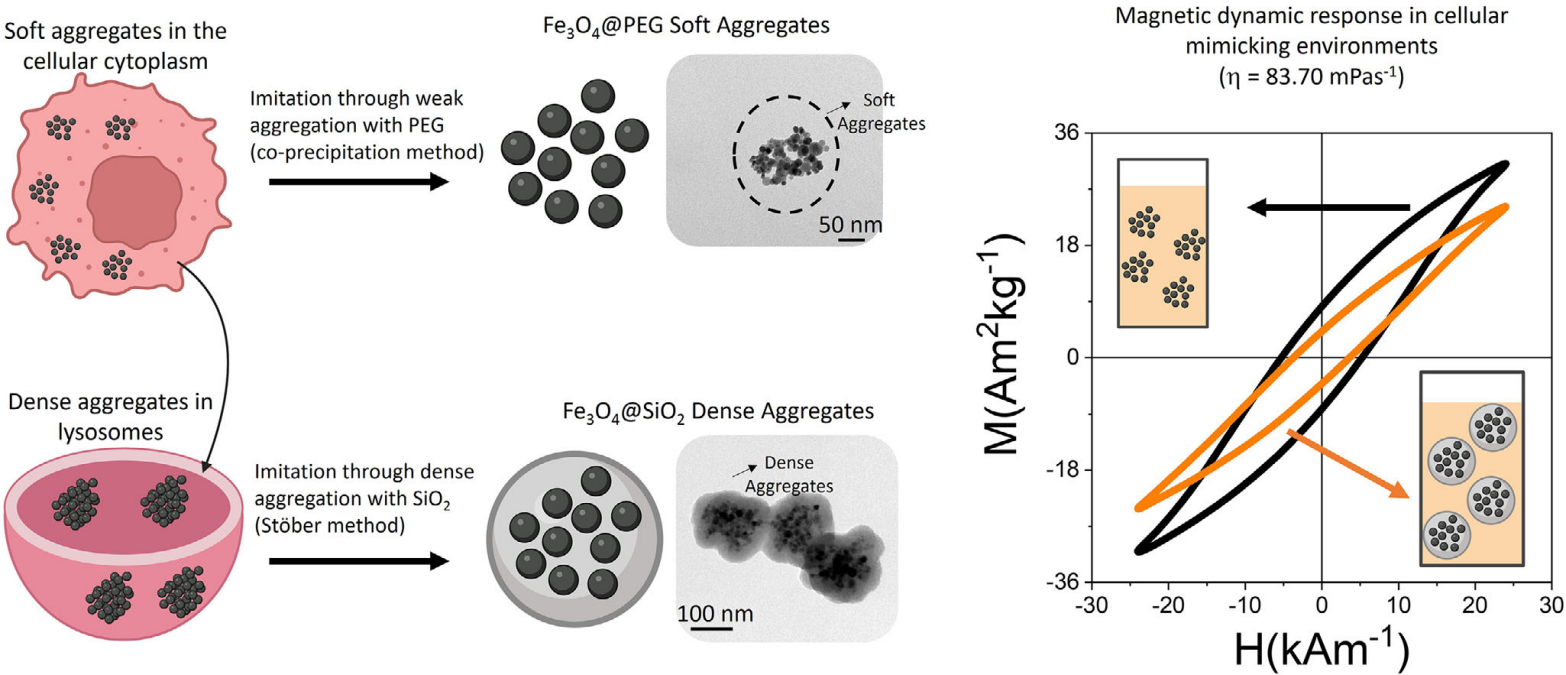
Schematic representation of the evaluation of the MH response of intracellular‐mimetic nanoparticle assemblies simulating ex situ cellular environments: Recent studies show that cells internalizing MNPs via endocytosis exhibit denser aggregation within lysosomes compared to other cellular regions.^[^ ^44^, ^54^ ^]^ Using synthetic techniques, the soft and reversible aggregation of MNPs (using PEG) and the dense and irreversible aggregation (in a SiO_2_ matrix) were simulated to evaluate their dynamic response in ex situ cellular‐mimicking environments (high viscosity media, η = 83.70 mPa·s).

This work presents an innovative approach to evaluate the effects of transitioning from soft to dense aggregation of iron oxide MNPs on MH efficiency, with the aim of experimentally mimicking cellular environments (**Figure** **1**). In contrast to previous works that examined aggregation induced by modifications in the surrounding medium, this study underscores the significance of invariant aggregation. The chemically induced aggregates were shown to accurately reproduce those observed within T lymphocytes and glioblastoma cells, both in size, morphology, and magnetic behavior. Their magnetic properties further revealed similarly strong dipolar interaction strengths, as confirmed by Henkel plot analysis, supporting the validity of the developed chemical aggregation model in mimicking natural intracellular clustering. The results highlight the dominant role of dense aggregation, showing a 20% decrease in MH efficiency compared to soft aggregates, in line with recent in vitro studies, and the negligible contribution of Brownian motion once aggregation is induced, with heat generation primarily governed by Néel relaxation. M_R_ is highlighted as a more reliable predictor and influencer of SAR in states of dense aggregation compared to H_C_, whose linear relationship with SAR, predicted by theoretical models, appears not to hold in high aggregation states. This underscores the key parameters that govern the efficiency of hyperthermia treatments in dense aggregates, which are responsible for the significant reduction of SAR during the internalization of MNPs in cellular media.

## 2 Results and Discussion

**Figure 2:**
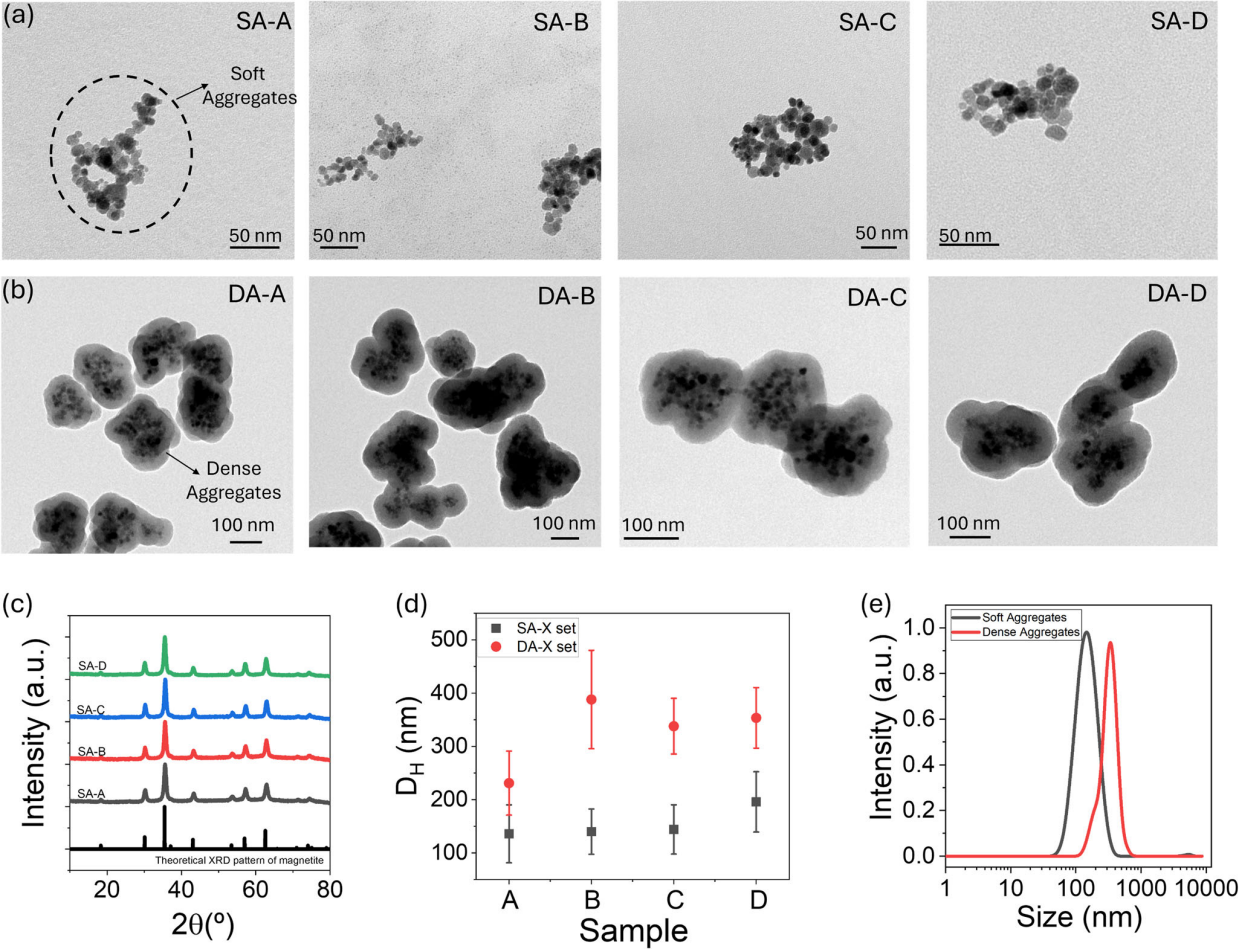
a) TEM micrographs of the soft aggregates (SA‐X set, top) and b) the dense aggregates (DA‐X set). c) XRD patterns of SA‐X set of MNPs: SA‐A (gray), SA‐B (red), SA‐C (blue), and SA‐D (green). d) D_H_ obtained by DLS for the soft aggregates (gray points) and dense aggregates (red points). e) Average D_H_ curve obtained for the soft (gray) and dense aggregates (red).

The induction of MNP aggregation through the described chemical procedures was analyzed using TEM micrographs (**Figure** **2**a,b). From these images, a spherical morphology of the soft aggregates (SA‐X MNPs), obtained via the co‐precipitation method, can be observed, revealing sizes ranging between 12–14 nm, as determined from the size distribution (Figure S1 and Table S1, Supporting Information). These images reveal the presence of irregular MNP aggregations, classified as soft aggregates, with an approximate size range of 100–150 nm. On the other hand, the dense aggregation of MNPs within SiO_2_ coatings, using the Stöber method, is evident from TEM micrographs (Figure 2b). In this case, clusters of MNPs ≈200 nm in size were observed, which, due to the inorganic nature of SiO_2_, exhibit consistent characteristics, denoted as dense aggregates (DA‐X MNPs). The presence of the SiO_2_ layer is observed as a light‐toned coating surrounding the magnetic cores, which appear darker, in irregular aggregates, resembling the irregular arrangement observed in soft aggregates. The similarity of the batches was demonstrated by consistent structural characterization and surface functionalization, which was critical for obtaining samples with different aggregation conditions while maintaining similar crystallinity. This ensured that the magnetic properties of the MNPs were not affected, despite the variations in aggregation states. X‐ray diffraction analysis revealed peaks corresponding to the inverse spinel structure of magnetite, indexed at (111), (220), (311), (400), (422), (511), (440), (620), and (553) (ICSD card No. 98‐015‐8742),^[^ ^55^ ^]^ indicating that, while the presence of maghemite cannot be ruled out, the predominant phase is magnetite (Figure 2c). Crystallite sizes, calculated using Scherrer's equation from the (311) peak, were found to be similar to or slightly smaller than the particle size, suggesting that each particle is likely a single crystal. Surface functionalization was assessed via FTIR spectroscopy (Figure S2, Supporting Information), where bands at 3400 cm^−1^ corresponded to O─H stretching of hydroxyl groups from adsorbed water molecules, and ≈550 cm^−1^ were attributed to the Fe^3^⁺–O^2−^ vibrations of Fe_3_O_4_ tetrahedral groups.^[^ ^56^ ^]^ Additional peaks at 344 and 1630 cm^−1^ were linked to ─OH stretching and asymmetric C═O vibrations, while the region ≈1050–1100 cm^−1^ indicated C─O bond vibrations. These findings confirm the successful attachment of PEG molecules to the surface of iron oxide MNPs. Finally, the magnetic performance of the obtained aggregates was demonstrated through DC magnetization loops (Figure S3, Supporting Information). Similar magnetic parameters were revealed across the four batches, with saturation magnetization (M_S_) values of ≈70 emu g^−1^ and superparamagnetic properties (M_R_ and H_C_ close to zero) within the range relevant for MH applications (T > 300 K). To confirm that differences in nanoparticle core size between batches S‐A and D did not significantly influence dipolar interaction effects, these interactions were quantified using Henkel plots. This approach, based on remanence measurements—specifically, isothermal remanent magnetization (IRM) and direct current demagnetization (DCD)—enables evaluation of dipolar interaction intensity.^[^ ^57^ ^]^ The presence of a negative peak in the Henkel plots (Figure S4, Supporting Information) confirms significant dipolar interactions, with an estimated average intensity of ≈66 ± 1% and no statistically significant differences between batches (maximum difference of only 2% between the highest and lowest values). These findings demonstrate that variations in core size did not alter the reproducibility of dipolar interaction strength. Comparison of the δM curves reveals that dense aggregates exhibit slightly deeper and broader minima (67 ± 1% on average), consistent with their more compact structure and enhanced local dipolar interactions. In contrast, soft aggregates show smoother and narrower δM responses, reflecting a more dispersed magnetic configuration. Thus, while aggregation density modulates the local interaction profile, the overall interaction intensity remains statistically similar between both types (Figure S4, Supporting Information). Additionally, to verify the reproducibility of the agglomeration process and the consistency of the magnetic and non‐magnetic phase fractions, the relative amounts of iron oxide and non‐magnetic constituents (PEG or silica) were determined by thermogravimetric analysis (TGA) combined with inductively coupled plasma optical emission spectroscopy (ICP‐OES). The analysis confirmed consistent phase composition across samples (Table S1, Supporting Information): soft agglomerates contained 5.3 ± 1.4 wt.% non‐magnetic material, whereas dense agglomerates contained 79.6 ± 1.8 wt.%, corresponding to magnetic‐to‐non‐magnetic mass ratios of 18:1 and 0.26:1, respectively. Collectively, these results validate the robustness of the synthetic protocol and confirm that the magnetic measurements were not biased by batch‐to‐batch variations.

The formation of aggregates in liquid media was verified by analyzing their dynamic properties, primarily hydrodynamic size (D_H_, Figure 2d) and polydispersity index (PDI, Figure S5, Supporting Information). Results demonstrated the development of soft aggregates driven by PEG‐mediated clustering. Notably, adjusting PEG concentration during synthesis resulted in an upward variation in D_H_, ranging from 135.9 to 195.8 nm in aqueous media, partially mimicking the variability of cell‐like aggregates. Conversely, dense aggregates, characterized by their encapsulation within an inorganic SiO_2_ shell, exhibited sizes close to 300 nm. The PDI values, consistently below 0.2 in all MNPs, indicated a high degree of uniformity among the aggregates. In summary, the average D_H_ reveals D_H_ = 142.6 nm (PDI = 0.138) for soft aggregates and D_H_ = 375.2 nm (PDI = 0.138) for dense aggregates (Figure 2e). The presence of the SiO_2_ coating on the dense aggregates accounts for this slight increase in size compared to the soft aggregates. Nonetheless, these results, in conjunction with the morphological observations from TEM micrographs, suggest that the aggregates generated by both synthetic approaches are well‐suited as model systems in terms of both size and dispersity.

**Figure 3:**
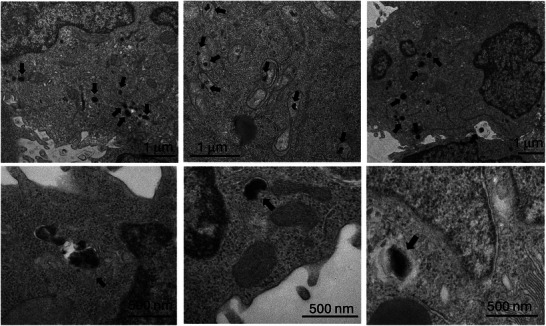
TEM images of ultrathin sections of living T lymphocytes treated with iron oxide nanoparticles at 100 µg mL^−1^ for 24 h incubation period. The arrows indicate electron‐dense structures that could represent lysosomal compartments potentially encapsulating nanoparticles, as well as irregular nanoparticle aggregates. Panels in the top row are shown at 1 µm scale; bottom row panels at 500 nm.

To explore the relationship between chemically induced aggregation and intracellular clustering, T lymphocytes were incubated with iron oxide nanoparticles (100 µg mL^−1^, 24 h), and the internalization and intracellular distribution of nanoparticles were examined by TEM (**Figure** **3**). Electron‐dense structures, distinguishable from the surrounding cytoplasm and absent in control cells (Figure S6, Supporting Information), were observed and are consistent with lysosomal compartments encapsulating nanoparticles. These compact aggregates, ranging from 82 to 301 nm (average: 174 ± 57 nm), likely correspond to nanoparticle clusters confined within vesicular compartments, potentially reflecting endocytic processing. Their morphology resembles that of chemically generated aggregates (Figure 2a,b), suggesting that vesicular confinement promotes interparticle interactions similar to those occurring in our model systems. Comparable intracellular nanoparticle clustering has been extensively reported in various cell types, where aggregates typically localize within lysosome‐like compartments ranging from 100 to 400 nm. For example, studies have shown progressive accumulation from small aggregates (<50 nm) to larger vesicular clusters (≈250 nm) in lymphoblastoid cells after 24 h,^[^ ^58^ ^]^ and similar patterns in stem cells and spheroids, where aggregate size and persistence strongly depend on particle coating, size, and exposure time.^[^ ^59^, ^60^, ^61^, ^62^ ^]^ In cancer cell lines such as HNE‐1, breast cancer, and A549 cells, marked intracellular aggregation within vesicular compartments of ≈200 nm has been observed, consistent with lysosomal localization and underscoring the relevance of this phenomenon in oncological contexts.^[^ ^63^, ^64^, ^65^, ^66^ ^]^ These findings collectively indicate that intracellular aggregation is a robust phenomenon, and in our case, the dense organization of nanoparticles within vesicles supports the presence of strong interparticle interactions.

**Figure 4:**
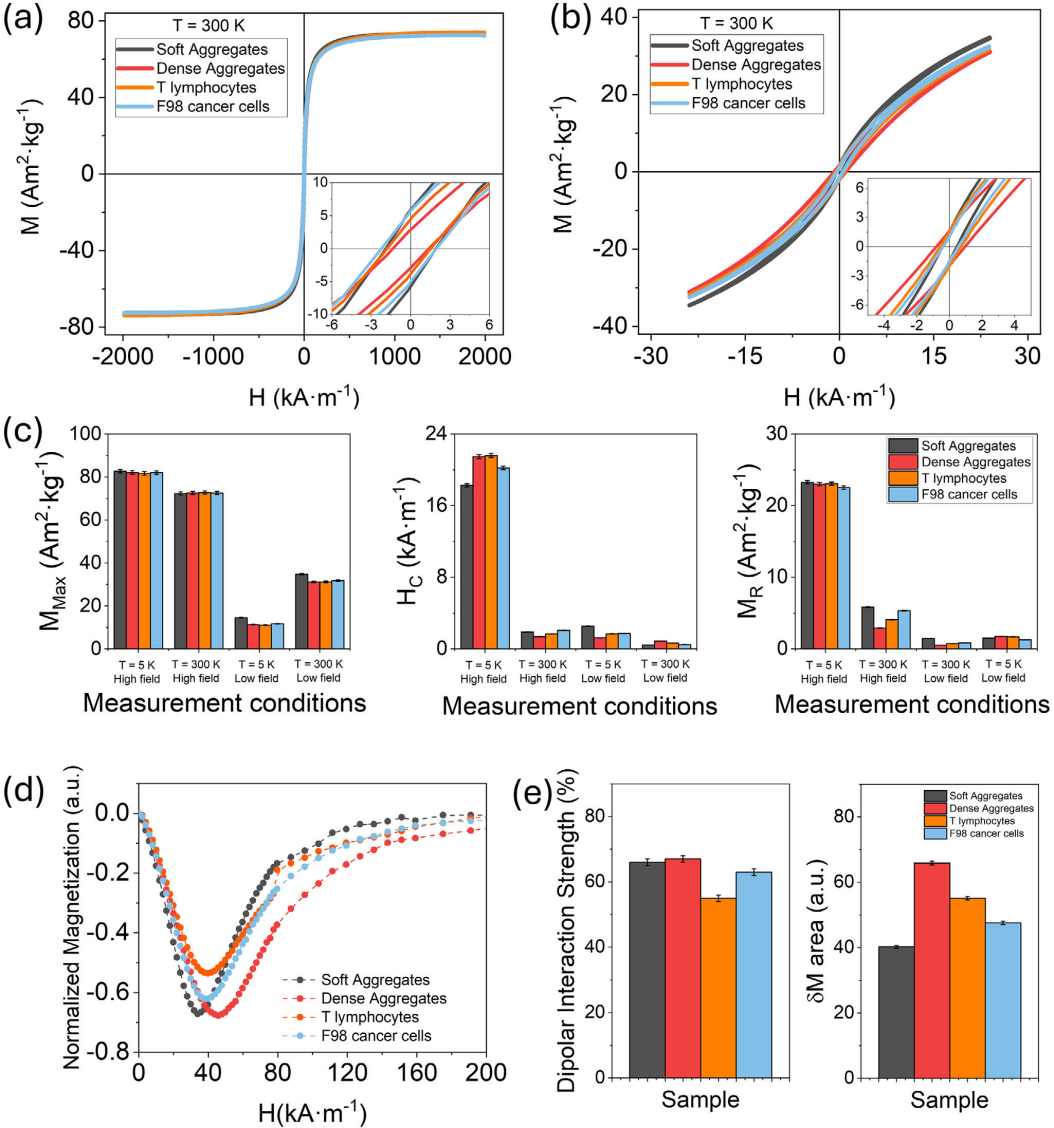
Magnetic hysteresis loops under DC conditions of chemically induced soft and dense aggregates and naturally occurring aggregates in T lymphocytes and F98 cancer cells, measured at T = 300 K under a) high‐field conditions (−2000 to +2000 kA m^−1^) and b) low‐field conditions (−24 to +24 kA m^−1^). c) Comparison of the magnetic parameters M_Max_, M_R,_ and H_C_ obtained from the hysteresis loops under different conditions. d) Representative Henkel plots for soft, dense, and naturally occurring aggregation in T lymphocytes and F98 cancer cells. e) Dipolar interaction strength (%) quantified from the maximum of the δM peak obtained via Henkel plot analysis (left) and the area under the curve of the δM plot (right). Colors denote aggregation types: soft aggregates (gray), dense aggregates (red), naturally induced aggregation in T lymphocytes (orange), and in cancerous F98 cells (blue). The same amount of magnetic material was employed (20 µg).

To assess the magnetic properties of naturally induced nanoparticle aggregates and their resemblance to those formed by chemical induction, T lymphocytes and F98 cancer cells were incubated with iron oxide nanoparticles (100 µg mL^−1^, 24 h). DC magnetic hysteresis loops recorded under high (±2000 kA m^−1^) and low (±24 kA m^−1^) fields, relevant for hyperthermia applications, showed a remarkable overlap between chemically induced aggregates—both soft and dense—and intracellular clusters (**Figure** **4**a,b; Figure S7, Supporting Information). Maximum magnetization values were similar across different types of aggregation, averaging 82.1 ± 0.5 A·m^2^·kg^−1^ at 5 K and 72.5 ± 0.3 A·m^2^·kg^−1^ at 300 K, indicating that the magnetic moment per iron oxide mass is largely independent of the aggregation state (Figure 4c). H_C_ at 5 K ranged from 18.3 kA·m^−1^ (soft aggregates) to 21.6 kA·m^−1^ (dense aggregates), with intracellular clusters falling in between (≈19.5–20.5 kA·m^−1^). At 300 K, H_C_ decreased sharply to 1.4–2.1 kA·m^−1^, maintaining the relative differences. M_R_ followed the same trend, ranging between 22.5 and 23.2 A·m^2^·kg^−1^ at 5 K, and dropping to 2.9–5.9 A·m^2^·kg^−1^ at 300 K, with values from aggregation occurring in cellular environments again falling between those of soft and dense aggregates. These observations reflect strong dipolar coupling and blocked magnetic moments at low temperature, transitioning toward superparamagnetic relaxation near physiological conditions. Analysis of Henkel plots, which probe dipolar interactions through irreversible magnetization, revealed some slight differences among the samples (Figure 4d). From this analysis, the dipolar interaction strength was obtained through the maximum of the δM peak, while the integrated area under the δM curve indicates the overall extent and distribution of these interactions (Figure 4e). Dense chemically induced aggregates exhibited the highest dipolar interaction strength (67 ± 1%) and the largest area under the δM curve (66 ± 1 a.u.), suggesting strong and widespread dipolar interactions. Soft aggregates showed a comparable dipolar interaction strength (66 ± 1%) but a smaller area (40 ± 1 a.u.), indicating more localized coupling. Intracellular clusters from T lymphocytes and F98 cells exhibited intermediate dipolar interaction strengths (55 ± 1% and 63 ± 1%, respectively) and areas (55 ± 1 a.u. and 48 ± 1 a.u., respectively), consistent with a heterogeneous mixture of aggregate densities and dipolar interaction strengths within the cellular environment. This heterogeneity aligns well with the hysteresis data, supporting the notion that chemically induced aggregates can effectively mimic the complex dipolar interactions present in intracellular nanoparticle clusters.

**Figure 5:**
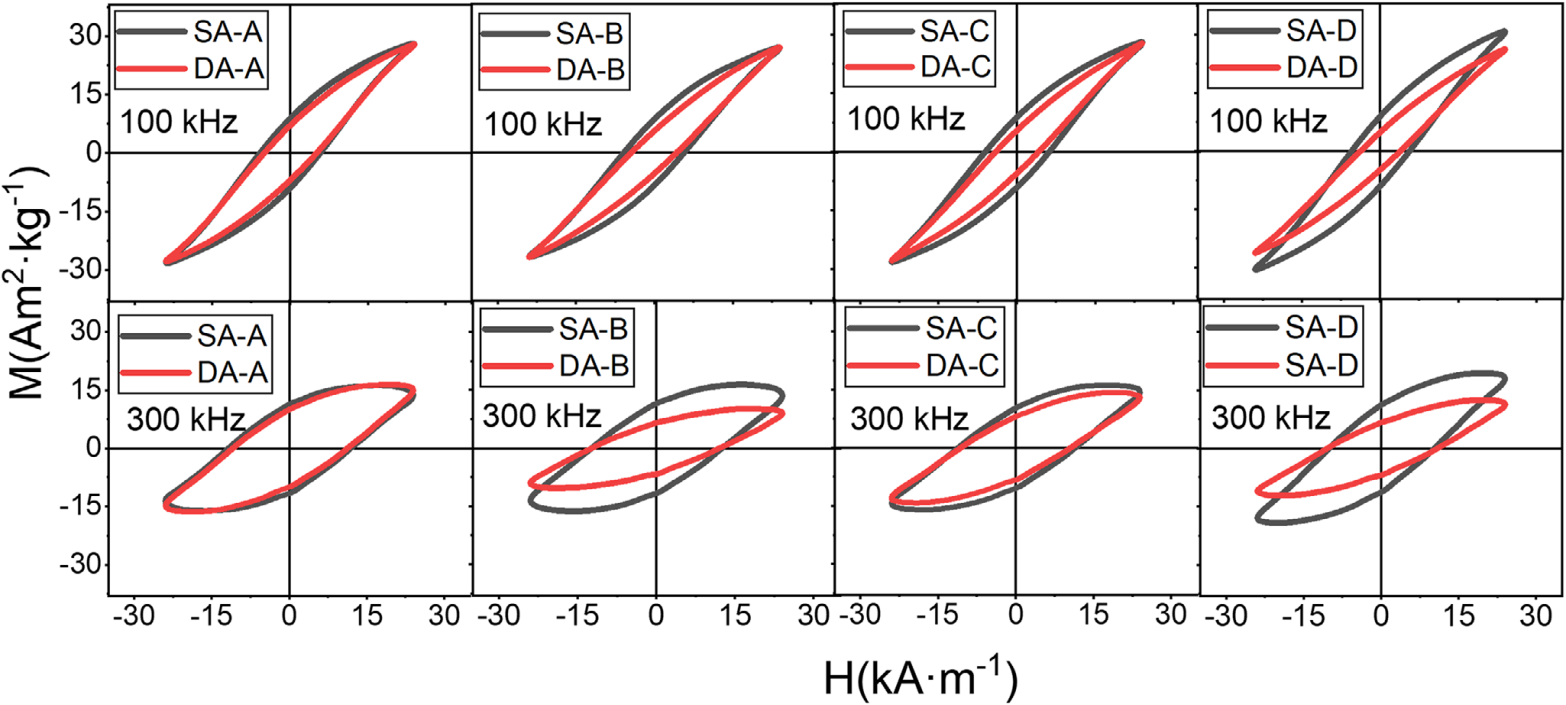
Hysteresis loops under AC conditions, 24 kA m^−1^ and 100 kHz (top) and 24 kA m^−1^ and 300 kHz (bottom) of the SA‐X set (black curves) and the corresponding aggregated DA‐X set (red curves). The MNPs were dispersed in water [Fe_3_O_4_] = 0.50 mg·mL^−1^, with a viscosity of η = 1.00 mPa·s.

To further understand how aggregation affects the dynamic response during hyperthermia, it is essential to consider how the spatial distribution of MNPs influences their magnetic behavior. The aggregation state of the MNPs will depend on the number of MNPs present in each volume, that is, on the concentration and, therefore, on the distance between the aggregates. The volumetric fraction of dispersion is the parameter that allows quantifying this distance between MNPs and is given by the ratio between the volume occupied by MNPs in the dispersion (V_MNP,d_) and the total dispersion volume (V_d_).^[^ ^38^ ^]^ In this sense, the higher the ϕ_d_, the closer the aggregates will be to each other, and the role of inter‐aggregate interactions will dominate. Therefore, to mimic the aggregation conditions of MNPs in biological environments, ϕ_d_ must be minimized, allowing the magnetic heating efficiency to be maximally influenced by intra‐aggregate interactions. In this context, a comparative study of the magnetic dynamic response of MNPs in states of soft aggregates and dense aggregates was carried out using a ϕ_d_ = 0.01 (corresponding to [Fe_3_O_4_] = 0.5 mg·mL^−1^). Initially, this dynamic response was investigated in low viscosity aqueous media (η = 1.00 mPs·s^−1^), respectively, employing two different frequencies, 100 and 300 kHz (**Figure** **5**). The results show similar trends, with magnetization loops of dense aggregates (red plots) exhibiting lower M_R_ and H_C_ compared to soft aggregates (black plots) in most cases, while M_Max_ is only reduced in specific cases. Additionally, significant differences in the dynamic response were observed depending on the frequency used, for both the soft and dense aggregates. At a frequency of f = 100 kHz, the rate‐dependent magnetization loops exhibit enhanced magnetization accompanied by reduced H_C_ and M_R_. Conversely, increasing the frequency to f = 300 kHz results in a decrease in magnetization, along with an increase in both H_C_ and M_R_.

However, due to experimental observations of the drastic reduction in SAR in biological environments (mainly in living cells), it becomes necessary to investigate the effect of aggregation in biological‐like environments, characterized by high viscosity. For this purpose, the magnetic dynamic response of the soft and dense aggregates was investigated in a highly viscous solution (η = 83.70 mPa·s, Figure S8, Supporting Information). This viscosity value was deliberately chosen because it lies within the range reported for intracellular compartments, particularly lysosomes—key sites for dense nanoparticle aggregation—where viscosities of ≈47 to 190 mPa·s have been documented.^[^ ^67^ ^]^ Therefore, this medium provides a physiologically relevant environment that closely mimics the biophysical constraints encountered within lysosomal compartments of cells. In both aggregate types, the frequency of the AC magnetic field was the dominant factor influencing the magnetization loops, while aggregate structure and medium viscosity contributed to a lesser extent. Nevertheless, the observed dependence on viscosity supports the relevance of studying magnetic heating under biologically realistic conditions, where intracellular environments such as lysosomes exhibit similarly high viscosities.

**Figure 6:**
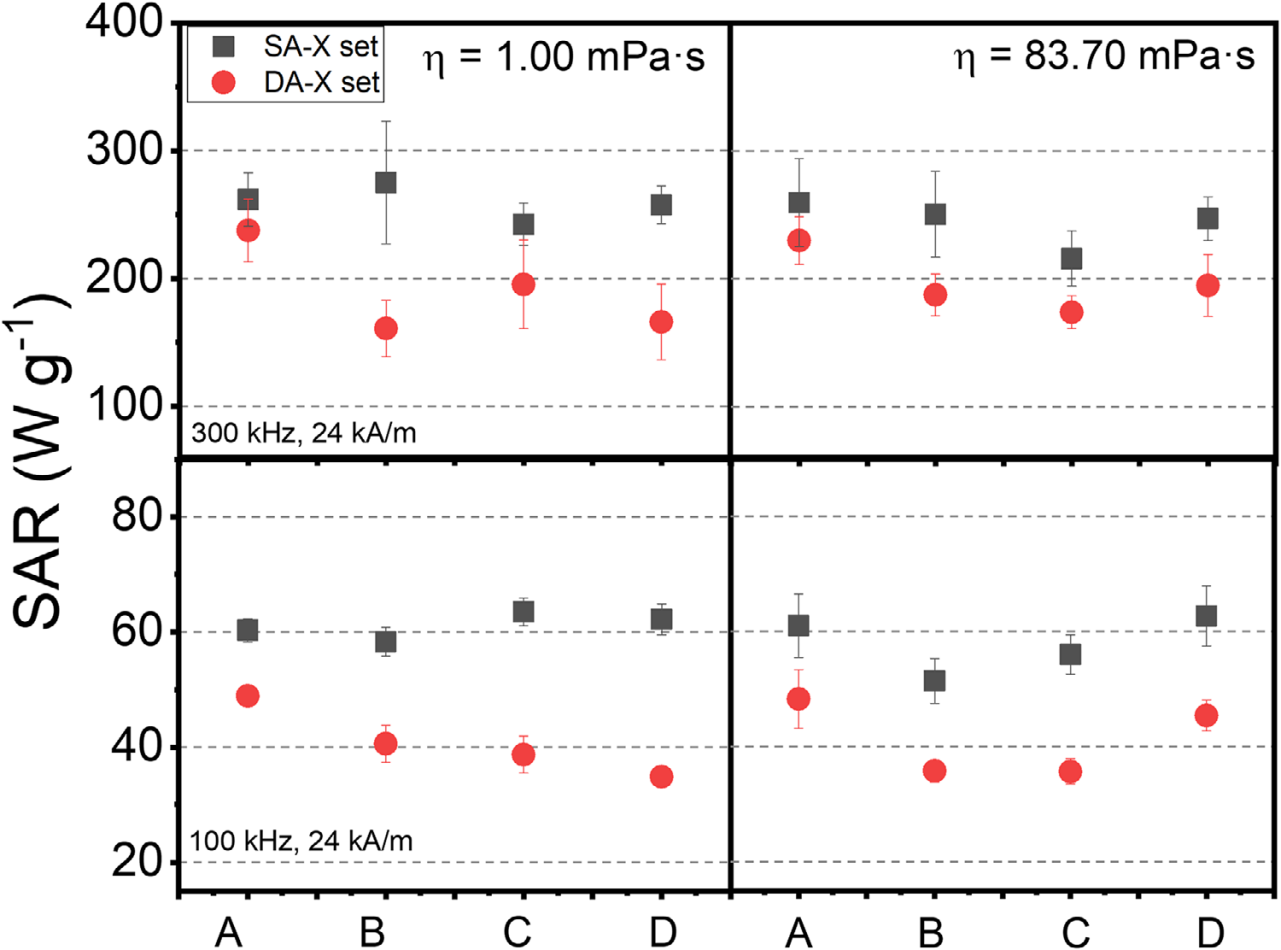
SAR values obtained from the dynamic response of soft aggregates (SA‐X set ‐ black dots) and dense aggregates (DA‐X set ‐ red dots) dispersed in low viscosity media (η = 1.00 mPs·a, left) and in high viscosity media (η = 83.70 mPa·s, right) at [Fe_3_O_4_]= 0.50 mg·mL^−1^.

A comparative analysis of the SAR values for soft (gray) and dense aggregates (red), obtained in aqueous and high‐viscosity media at [Fe_3_O_4_] = 0.5 mg·mL^−1^, was performed to observe these differences more precisely (**Figure** **6**). The observed results revealed lower SAR values in the dense aggregates across all batches, though with slight variations. In aqueous media, SAR reduction appears to be greater compared to high‐viscosity media, where very similar values between soft aggregates and dense aggregates can be observed. Specifically, SAR values at f = 300 kHz are generally above 200 W·g^−1^, while in dense aggregates they decrease below 200 W·g^−1^. Similarly, at f = 100 kHz, soft aggregates showed values ≈60 W·g^−1^ compared to ≈40 W·g^−1^ for dense aggregates.

**Figure 7:**
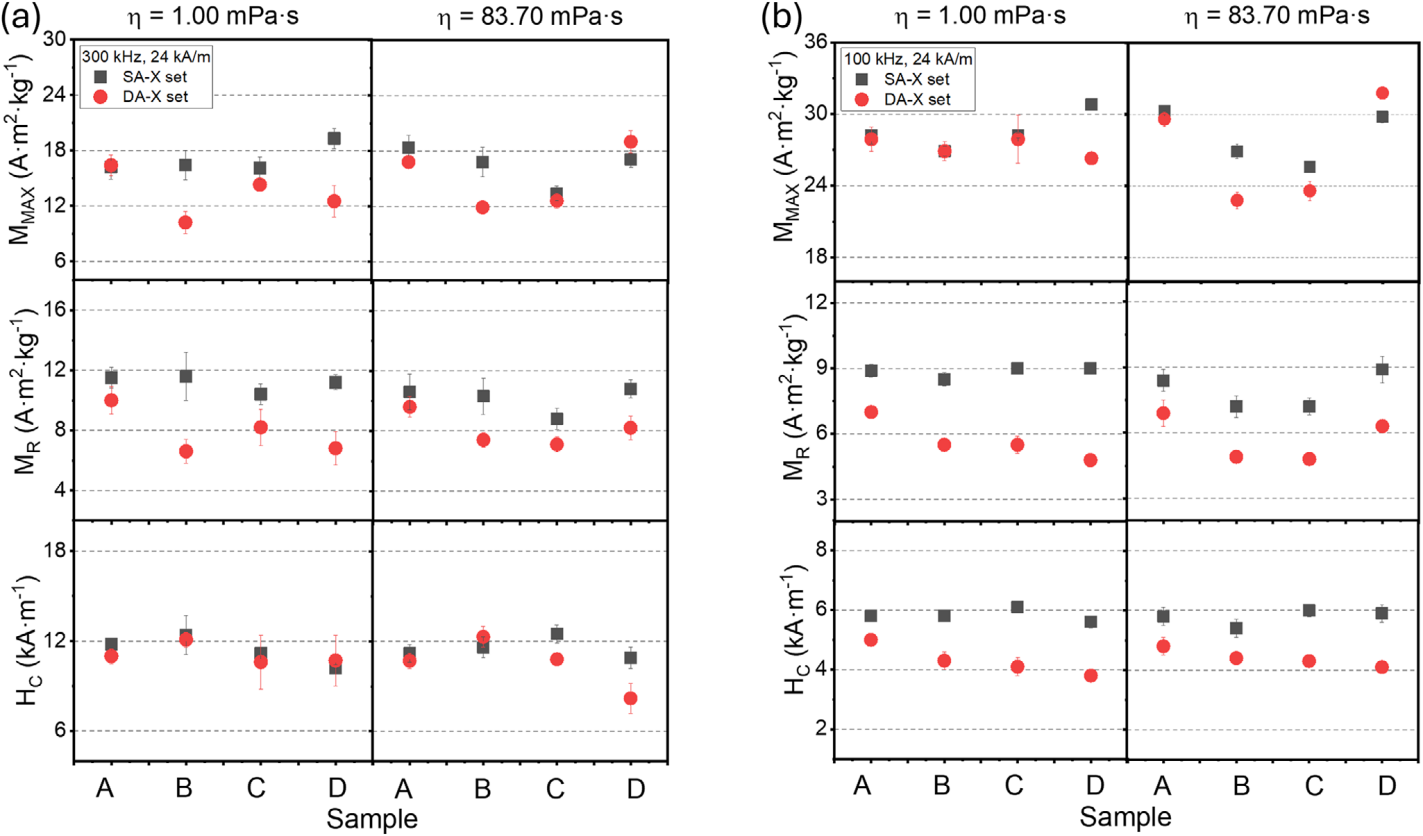
H_C_, M_R_ and M_Max_ values from the dynamic response of soft aggregates (SA‐X set ‐ black dots) and dense aggregates (DA‐X set ‐ red dots) dispersed in low viscosity media (η = 1.00 mPa·s) and in high viscosity media (η = 83.70 mPa·s) at [Fe_3_O_4_] = 0.50 mg·mL^−1^ and exposed to a) high frequency (f=300 kHz), and b) low frequency (f=100 kHz).

Since the SAR in the present study corresponds to the magnetic losses obtained from the area of the magnetization loops, it depends on H_C_, M_R_, and M_Max_, which in turn depend on the material characteristics of the soft and stiff aggregates. A comparative analysis of H_C_, M_R_, and M_Max_ between soft (black) and dense aggregates (red), both in aqueous and high‐viscosity media, was performed (**Figure** **7**). The results revealed that the main differences were observed for H_C_ and M_R_, while the M_Max_ values showed minimal decrease. On the other hand, M_R_ did not show a clear trend, with consistently lower values in the case of dense aggregates. More specifically, under conditions of η = 1.00 mPa·s, the H_C_ values at 300 kHz for the SA‐X set are ≈12 kA m^−1^ across all samples (A, B, C, D), whereas the DA‐X set shows slightly lower H_C_ values. At f = 100 kHz, both sets exhibit reduced H_C_ values, with the SA‐X set ≈6 kA m^−1^ and the DA‐X set ≈4 kA m^−1^, demonstrating greater differences than at a higher frequency. In cell‐mimicking media with increased viscosity (η = 83.70 mPa·s) the trend observed was similar, with H_C_ values at f = 300 kHz for the SA‐X set rising to ≈12 kA m^−1^, while the DA‐X set remains slightly lower. At f = 100 kHz, the SA‐X set shows H_C_ values of ≈7‐8 kA m^−1^, and the DA‐X set ≈4 kA m^−1^. These results demonstrate that higher H_C_ values are consistently observed at f = 300 kHz compared to f = 100 kHz for both sample sets. Overall, the SA‐X set consistently exhibits higher H_C_ values compared to the DA‐X set under all tested conditions.

The M_Max_ parameter was not significantly affected by adding in dense packing situations. There is a slight increase in M_Max_ for SA‐X set as the viscosity increases from 1.00 to 83.70 mPa·s. In addition, the DA‐X set shows similar trends but with generally lower M_Max_ compared to the SA‐X set. A similar trend of increasing M_Max_ with increasing viscosity was observed. Regarding the M_R_ results, more pronounced differences were observed, which could play a key role. M_R_ was higher in the SA‐X set compared to the DA‐X set across all samples (A, B, C, D). The transition from aqueous to cell‐mimicking media leads to a slight decrease in M_R_ for the SA‐X set and a more significant decrease for the DA‐X set. In summary, both M_Max_ and M_R_ show consistent values across different samples (A, B, C, D) within each experimental condition, indicating reproducibility. Increased viscosity leads to an increase in M_Max_ for both SA‐X and DA‐X sets, potentially due to enhanced alignment of MNPs under higher viscous drag. Conversely, M_R_ decreases with increased viscosity, suggesting that higher viscosity may hinder the ability of magnetic moments to remain aligned after the external field is removed. Soft aggregates consistently exhibit higher M_Max_ and M_R_ values compared to dense aggregates, suggesting more favorable magnetic properties for both parameters. In contrast, the lower magnetization values observed in dense aggregates could be attributed to stronger dipolar interactions and enhanced magnetic anisotropy.

**Figure 8:**
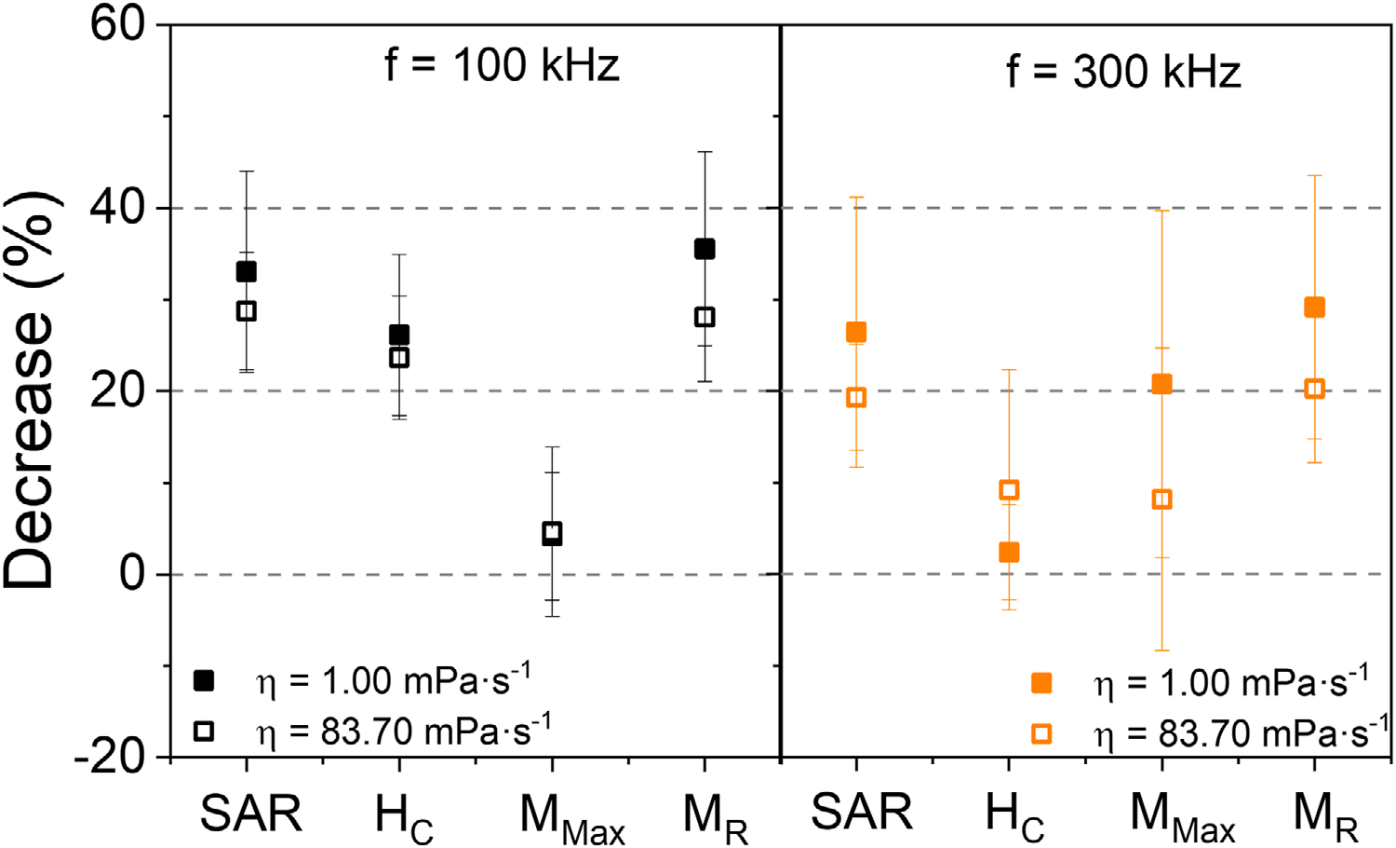
Percentage decrease in the SAR, H_C_, M_Max_, and M_R_ values of dense aggregates compared to soft aggregates in low viscosity media (close dots) and high viscosity media (open dots) at f = 100 kHz (left) and f = 300 kHz (right).

The reductions observed in the various parameters were quantified as the ratio of the values of soft aggregates to the values of dense aggregates, to obtain an average percentage reduction across different batches (**Figure** **8**). Generally, a greater reduction was observed at a lower frequency (f = 100 kHz), with the SAR reduction percentage exceeding 20% in all cases, demonstrating the drastic decrease caused by the dense aggregation of MNPs. Among the influential parameters, M_R_ was revealed to play the most significant role, consistently showing a reduction exceeding 20%, regardless of frequency and viscosity. The other relevant parameter, H_C_, also showed a notable influence on SAR reduction, although in many cases the reduction did not surpass 20%. Specifically, a reduction of H_C_ of at least 20% is observed at f = 100 kHz. However, at f = 300 kHz, this reduction is below 20%, with some values approaching zero. M_Max_ was found to have a residual influence, as no drastic reductions were observed, indicating that aggregation states do not alter the magnetization of the systems. For f = 300 kHz, a reduction in M_Max_ of ≈20% was observed, although this could be attributed to a modification of colloidal properties following the application of alternating magnetic fields.

These results demonstrate that the effect of aggregation, regardless of whether the aggregation is loose or dense, shows more similarity in environments where the motion of MNPs is highly restricted (high‐viscosity environments). In such restricted environments, the influence of Brownian relaxation becomes increasingly negligible, as the viscous environment limits the physical rotation and movement of individual MNPs. Due to the aggregation and immobilization of MNPs, particularly within dense aggregates, the system may approach a state resembling infinite viscosity. This suggests that in high‐viscosity conditions, the contribution of Brownian motion to the overall response of the system is significantly reduced, and the properties are more governed by other mechanisms, such as Néel relaxation. To validate the proposed hypothesis, the dynamic magnetic response of both soft and dense aggregates was evaluated under conditions of complete immobilization (solid state) and compared to that in a high‐viscosity medium (Figure S8, Supporting Information). Magnetization hysteresis loops measured under AC conditions (top panels) exhibit only marginal differences between the two environments, particularly for dense aggregates (Figure S9, Supporting Information). This negligible variation could indicate that Brownian relaxation was effectively suppressed in viscous media, especially within aggregated systems. Key quantitative parameters, including SAR, M_R_, and H_C_, were analyzed (bottom panels, Figure S9, Supporting Information). These metrics show minimal variation between high‐viscosity and solid‐state conditions, remaining within experimental uncertainty. Importantly, no significant decline in SAR efficiency was observed upon solidification, and differences were even less pronounced for DA samples, where increased structural compactness further restricts rotational freedom. This consistent behavior reinforces the interpretation that Néel relaxation predominates under these conditions, with Brownian relaxation playing a negligible role in aggregation states, both soft and especially dense. However, the degree of aggregation could play a significant role, as a greater reduction in SAR was observed. The primary reason for this may lie in the effects of MNP interactions, which impact the anisotropy constant^[^ ^68^ ^]^ and, consequently, influence variations in Néel relaxation. Additionally, these results demonstrate that, despite the importance attributed to the effect of H_C_ on SAR reduction, it appears that M_R_ predominates in this reduction within dense aggregates. During the last years, some empirical relationships have been established between the relevant parameters, M_R_, M_Max_, H_C_, and SAR. Specifically, through numerical simulations, for a MNP with its easy axis aligned along the magnetic field direction, the area of this hysteresis loop is maximal and is given as:^[^ ^69^ ^]^

(1) $$\mathrm{SAR}=A\cdot f=4\mu_{o}H_{C}M_{S}f=8\;K_{\mathrm{eff}}\;$$

However, in the case of randomly oriented MNPs, more accurate for experimental conditions, this area is reduced to:^[^ ^69^ ^]^

(2) $$\mathrm{SAR}=A\cdot f=2\mu_{o}H_{C}M_{S}f=1.92\;K_{\mathrm{eff}}$$

**Figure 9:**
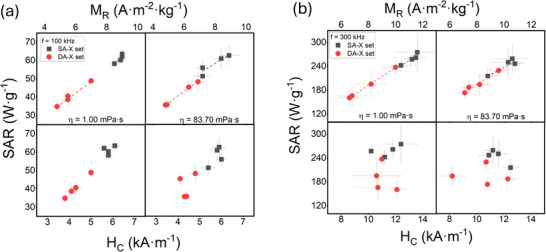
Dependency of SAR on H_C_ (bottom) and M_R_ (top) for soft aggregates (black dots) and dense aggregates (red dots) at 0.50 mg·mL^−1^, and in media of different viscosities: 1.00 and 83.70 mPa·s. The measurements were taken under the application of a 24 kA·m^−1^ field at a frequency of a) 100 kHz and b) 300 kHz.

In this context, the relationship between SAR and magnetic properties—M_R_ and H_C_ was experimentally investigated at two different frequencies: 100 kHz (**Figure** **9**a) and 300 kHz (Figure 9b). Both plots present data for two sample types: soft aggregates (black squares) and dense aggregates (red circles), measured under varying viscosity conditions. A positive correlation between SAR and M_R_ was observed for both frequencies and viscosities. SAR increases linearly with M_R_, indicating that higher M_R_ leads to greater energy absorption. At higher viscosity, η = 83.70 mPa·s, the positive correlation remains, though the slope is slightly less steep, indicating that higher viscosity may dampen the increase in SAR with M_R_. Interestingly, this linear correlation between M_R_ and SAR appears to persist throughout the transition from soft to dense aggregates, suggesting that M_R_ may serve as a sensitive indicator of the internal structural evolution of nanoparticle assemblies. In particular, intermediate M_R_ values in samples transitioning between the soft and dense aggregate states are accompanied by proportionally intermediate SAR values, supporting the notion of a gradual, rather than abrupt, change in magnetic dissipation behavior as aggregation increases. This implies that M_R_ captures not only the intrinsic magnetic response but also the collective magnetic behavior influenced by interparticle interactions and structural compaction within the aggregates. Conversely, no clear linear relationship was observed between SAR and H_C_. A linear correlation was only evident for soft aggregates at f = 100 kHz in aqueous media (η = 1.00 mPa·s), whereas under all other conditions, the relationship appeared random. These results suggest that although H_C_ contributes to energy absorption, additional factors also influence the SAR values. In summary, these results suggest that M_R_ emerges as a more reliable parameter for predicting the heating efficiency of MNPs in aggregated states compared to H_C_. The linear relationship between M_R_ and SAR, especially in dense aggregates, implies that M_R_ plays a significant role in the energy absorption capacity of MNPs under alternating magnetic fields, in accordance with the proposed models. This predictive capability means that M_R_ could also serve as a sensitive indicator not only for evaluating heating efficiency but also for detecting potential aggregation‐induced magnetic clustering that may adversely affect cellular uptake and therapeutic efficacy of MNPs. Conversely, the absence of a clear relationship between H_C_ and SAR indicates that coercive field strength may be influenced by other factors in aggregated states, such as dipolar interactions among particles, breaking the theoretically established predictions. This scattering could be attributed to the complex magnetic interactions and the structural arrangement of MNPs within the aggregates, which affect H_C_ differently than M_R_.

## 3 Conclusion

The work offers valuable insights into the aggregation behavior of MNPs by developing intracellular‐mimetic nanoparticle assemblies, simulating cellular environments, and evaluating the transition from soft to dense aggregation states in terms of MH efficiency. To achieve this, the aggregation of ≈10 nm MNPs was implemented using soft coatings based on PEG polymeric matrices and stiff coatings based on inorganic SiO_2_ matrices. The aggregates exhibited sizes ranging from 100–200 nm for soft aggregates and ≈200 nm for dense aggregates. It was demonstrated that these chemically induced nanoparticle aggregates closely resemble those naturally formed in biologically relevant cells such as T lymphocytes and glioblastoma (F98) cells. The similarity in size, morphology, and magnetic interactions — particularly the demonstrated strong dipolar interactions that play a key role — supports the validity of the model system for studying intracellular aggregation and its impact on magnetic hyperthermia. The dynamic response was evaluated in both low‐viscosity and high‐viscosity environments (mimicking different cellular conditions) and demonstrated that, once aggregation is induced, the role of viscosity becomes practically negligible. Stiff aggregation was found to induce a reduction in MH efficiency of over 20% and, in general, M_R_ was identified as the key parameter in this reduction, followed by H_C_ with a secondary role, M_Max_ was deemed negligible. Moreover, the dependence of SAR, and thus MH efficiency, on these parameters was evaluated in MNP aggregates. It was shown that M_R_ modulates SAR following a linear dependence, as predicted by various models, while the dependence on H_C_ appeared random, contradicting theoretical expectations and highlighting the relevance of aggregation in modulating H_C_. This suggests that M_R_ is a more reliable predictor of heating efficiency, particularly at higher frequencies. Consequently, enhancing M_R_ in MNPs will be crucial for improving their performance in MH applications. By focusing on improving M_R_ and understanding the complex interplay between aggregate structure, medium viscosity, and magnetic properties, future research can pave the way for significant advancements in the field of nanomedicine.

## 4 Experimental Section

### Chemicals

All chemicals used in the synthetic processes were used as received without further purification. Iron(III) chloride hexahydrate (FeCl_3_·6H_2_O, 99%), iron(II) sulphate heptahydrate (FeSO_4_·7H_2_O, 99%), poly(ethylene glycol) (PEG, C_2n_H_4n + 2_O_n + 1_, Mw: 300), tetraethyl orthosilicate (TEOS, SiC_8_H_20_O_4_, 98%), ammonium hydroxide (NH_3_ aq, 28%), and hydrochloric acid (HCl, 37%) were purchased from MERCK (Saint Louis, MO, USA). Ethanol (C_2_H_5_OH, absolute grade) was purchased from Panreac (Madrid, Spain). Milli‐Q (Millipore, Burlington, MA, USA) deionized water was used in all the experiments.

### Synthesis of Variant Soft‐Aggregates of Magnetite Nanoparticles (Fe_3_O_4_@PEG)

The synthesis of soft MNPs aggregates was carried out using the coprecipitation method, employing PEG as the encapsulating reagent for the MNPs. Briefly, iron chloride hexahydrate (45 mmol) and iron (II) sulphate heptahydrate (30 mmol) were dissolved in 100 mL of 10 mm HCl aqueous solution through mechanical stirring. The mixture was heated to 60 °C and then NH_3_ aq (770 mmol) and the corresponding amount of PEG (M_W_ = 300) were added. Different percentages of PEG with respect to the initial volume were used, 5, 10, 20, and 30%. Then, the reaction was cooled to room temperature and subsequently acidified to pH 5 with the addition of HCl (1 m), in order to remove any remaining unreacted salts. Then, the obtained MNPs were washed 6 times with milli‐Q water by separation with permanent magnets. The MNPs obtained were denoted as “SA‐X” (Soft‐aggregates), with X = A, B, C, or D depending on the percentage of PEG used in the synthetic procedure.

### Synthesis of Dense Invariant Aggregates of Magnetite Nanoparticles (Fe_3_O_4_@SiO_2_)

The dense clustering of MNPs was achieved through the encapsulation of Fe_3_O_4_@PEG MNPs (SA‐X MNPs) within inorganic SiO_2_ shells. In this procedure, the TEOS precursor facilitated the seed‐mediated growth of silica onto the surface of magnetite nanoparticles previously functionalized with PEG via the co‐precipitation method. Initially, 1 mL of SA‐X MNPs (3.5 mg mL^−1^) was dispersed in ethanol (5 mL) by sonication in a bath for 15 min to ensure uniform dispersion. Subsequently, NH_3_ aq (114.6 µL) and TEOS (100 µL) were added to the mixture and stirred orbitally using an incubator at room temperature for 24 h. Following this incubation period, the MNPs were separated magnetically, subjected to washing steps, and subsequently redispersed in Milli‐Q water. The MNPs obtained were denoted as “DA‐X” (Dense aggregates), with X = A, B, C, or D depending on the percentage of PEG used in the synthetic procedure.

### Physicochemical Characterization

The crystalline phases were characterized using X‐ray diffraction (XRD) with powder samples employing a Philips PW1710 diffractometer (Panalytical, Brighton, UK) equipped with a Cu Kα radiation source (λ = 1.54186 Å). Measurements were collected within the 2θ angle range of 10° to 80° with steps of 0.02° and a duration of 10 s/step. The peak broadening observed in the XRD patterns was utilized to determine crystallite size utilizing Scherer's equation. Morphology and size analysis of the MNPs were conducted via transmission electron microscopy (TEM) using a JEOL JEM‐1011 microscope operating at 100 kV (JEOL, Tokyo, Japan). Samples were prepared on copper grids with Formvar films for analysis. The Image J software (distributed by NIH, USA) was employed to measure the diameters of the MNPs. Fourier transform infrared (FTIR) spectra of the surface functional groups of the nanostructures were recorded using a Thermo Nicolet Nexus spectrometer (Thermo Fisher Scientific, Madrid, Spain) employing the attenuated total reflectance (ATR) method over the range of 4000 to 400 cm^−1^. Measurements of hydrodynamic particle size and ζ‐Potential of MNPs were conducted using a Zetasizer Nano ZS (Malvern Instruments, Worcestershire, UK) equipped with a He–Ne laser (633 nm). The analysis was performed at a scattering angle of 173° and at room temperature. All analyses were conducted in triplicate. Quantification of Fe and Si was performed by inductively coupled plasma optical emission spectroscopy (ICP‐OES, Perkin‐Elmer Optima 3300 DV).The composition of the samples was analyzed with a TGA Perkin Elmer model 7 (Perkin Elmer, Waltham, MA, USA).

### In vitro assays

Primary human T lymphocytes and F98 glioblastoma cells were selected for the in vitro assays. T lymphocytes were derived from peripheral blood mononuclear cells (PBMCs) isolated from buffy coats by Histopaque density gradient centrifugation. PBMCs were cultured in RPMI 1640 medium supplemented with 10% fetal bovine serum (FBS) and 1% penicillin‐streptomycin (P/S), and stimulated with phytohemagglutinin (PHA, 1 µg mL^−1^) for 48 h at 37 °C and 5% CO_2_. Afterward, cells were expanded into medium containing IL‐7 (400 IU mL^−1^) and IL‐15 (200 IU mL^−1^) until day 6. Non‐adherent T cells were collected, counted, and seeded at 1 × 10⁶ cells per well in 24‐well plates with complete medium and cytokines. Nanoparticles were added at a final concentration of 100 µg mL^−1^, and cells were incubated for 24 h. F98 glioblastoma cells were cultured in DMEM supplemented with 10% FBS and 1% P/S under standard conditions (37 °C, 5% CO_2_). Cells were thawed and expanded until the day of the assay, when they were seeded in 24‐well plates and exposed to nanoparticles (100 µg mL^−1^) for 24 h. After incubation, T lymphocytes were processed for TEM. Cells were washed with PBS and fixed with 2.5% glutaraldehyde and 2.5% paraformaldehyde in 0.1 m cacodylate buffer (pH 7.2–7.4) for 2 h at room temperature or overnight at 4 °C. After washing with cacodylate (3x), samples were post‐fixed in 1% osmium tetroxide in cacodylate buffer for 1 h, dehydrated through graded ethanol series, and infiltrated with epoxy resin (Epon or Araldite). The blocks were polymerized at 60 °C for 24–48 h. Ultrathin sections (70–90 nm) were cut using an ultramicrotome, mounted on copper grids, stained with uranyl acetate and lead citrate, and imaged using a JEOL 1011 transmission electron microscope.

### Magnetic Characterization

Direct current (DC) magnetization curves of dried samples were measured using Superconducting Quantum Interference Device (SQUID) Magnetometer (Quantum Design, Darmstadt, Germany). Magnetic hysteresis loops at 300 K and 5 K were obtained under two different external magnetic field ranges: low fields ranging from −24 to 24 kA·m^−1^ and high fields from −2000 to 2000 kA·m^−1^. Isothermal remanent magnetization (IRM) and direct current demagnetization (DCD) measurements were performed at 5 K after thermal demagnetization. These data were then used to construct the Henkel plots:

(3) $$\delta M\left(H\right)=M_{D}\left(H\right)-\left[M_{\mathrm{RS}}-2M_{R}\left(H\right)\right]$$

where M_D_ is the DC demagnetization, M_RS_ is the saturation remanence, M_R_ is the isothermal remanence magnetization, and H is the applied magnetic field. For the magnetic measurements in the cellular assays, primary human T lymphocytes and F98 glioblastoma cells containing internalized nanoparticles were processed. Following the internalization procedure described in the previous methodology section, a pellet containing an amount of nanoparticles equivalent to 20 µg of magnetic material was isolated and used for the measurements in the SQUID magnetometer. AC magnetometry measurements were performed using a commercial inductive magnetometer (AC Hyster Series; Nanotech Solutions, Madrid, Spain) with the application of a magnetic field of 24 kA m^−1^ at frequencies of 100 and 300 kHz. For the development of the measurements in both liquid and solid media, the same amount of magnetic material was employed (20 µg).

### Statistical Analysis

All statistical analyses were performed using OriginPro 2016 (OriginLab Corporation, Northampton, MA, USA). Particle size distributions obtained from TEM images were analyzed using ImageJ and presented as mean ± standard deviation (SD); additionally, the Distribution Fit tool in OriginPro was applied to assess the goodness of fit of the particle size distributions. Measurements of hydrodynamic size, ζ‐potential, iron content, and AC hysteresis were carried out in triplicate (n = 3) and expressed as mean ± SD. Data from AC hysteresis measurements were carried out in triplicate (n = 3) and additionally processed using the Manyetic proprietary software (Nanotech Solutions, Madrid, Spain).

## Conflict of Interest

The authors declare no conflict of interest.

## Supporting information



Supporting Information

## Data Availability

The data that support the findings of this study are available from the corresponding author upon reasonable request.
