# Supplementary material for: Predicting the Dominant Role of Dense Aggregates in Magnetic Hyperthermia via Intracellular‐Mimetic Nanoparticle Models
Source: Small. 2025 Aug 29;21(41):e06620. doi: 10.1002/smll.202506620 (PMC12530015; doi:10.1002/smll.202506620)
Supplement: Supplementary file 1 — Supporting Information [file SMLL-21-e06620-s001.docx]

Supporting Information

Predicting the Dominant Role of Dense Aggregates in Magnetic Hyperthermia Via Intracellular-Mimetic Nanoparticle Models

Pelayo García-Acevedo*, Alba Paz-Castro, Jorge Estébanez, Yolanda Piñeiro and José Rivas*

Pelayo García-Acevedo, Alba Paz-Castro, Jorge Estébanez, Yolanda Piñeiro and José Rivas

NANOMAG Laboratory, Applied Physics Department, iMATUS Materials Institute and Health Research Institute of Santiago de Compostela (IDIS), Universidade de Santiago de Compostela, 15782, Santiago de Compostela, Spain.

E-mail: pelayo.garcia.acevedo@usc.es, jose.rivas@usc.es

Pelayo García-Acevedo

Neuroimaging and Biotechnology Laboratory (NOBEL), Clinical Neurosciences Research Laboratory (LINC), Health Research Institute of Santiago de Compostela (IDIS), Santiago de Compostela, Spain

E-mail: pelayo.garcia.acevedo@sergas.es

Alba Paz-Castro

Department of Biochemistry and Molecular Biology, Faculty of Biology-Biological Research Centre (CIBUS), Universidade de Santiago de Compostela, Santiago de Compostela, Spain


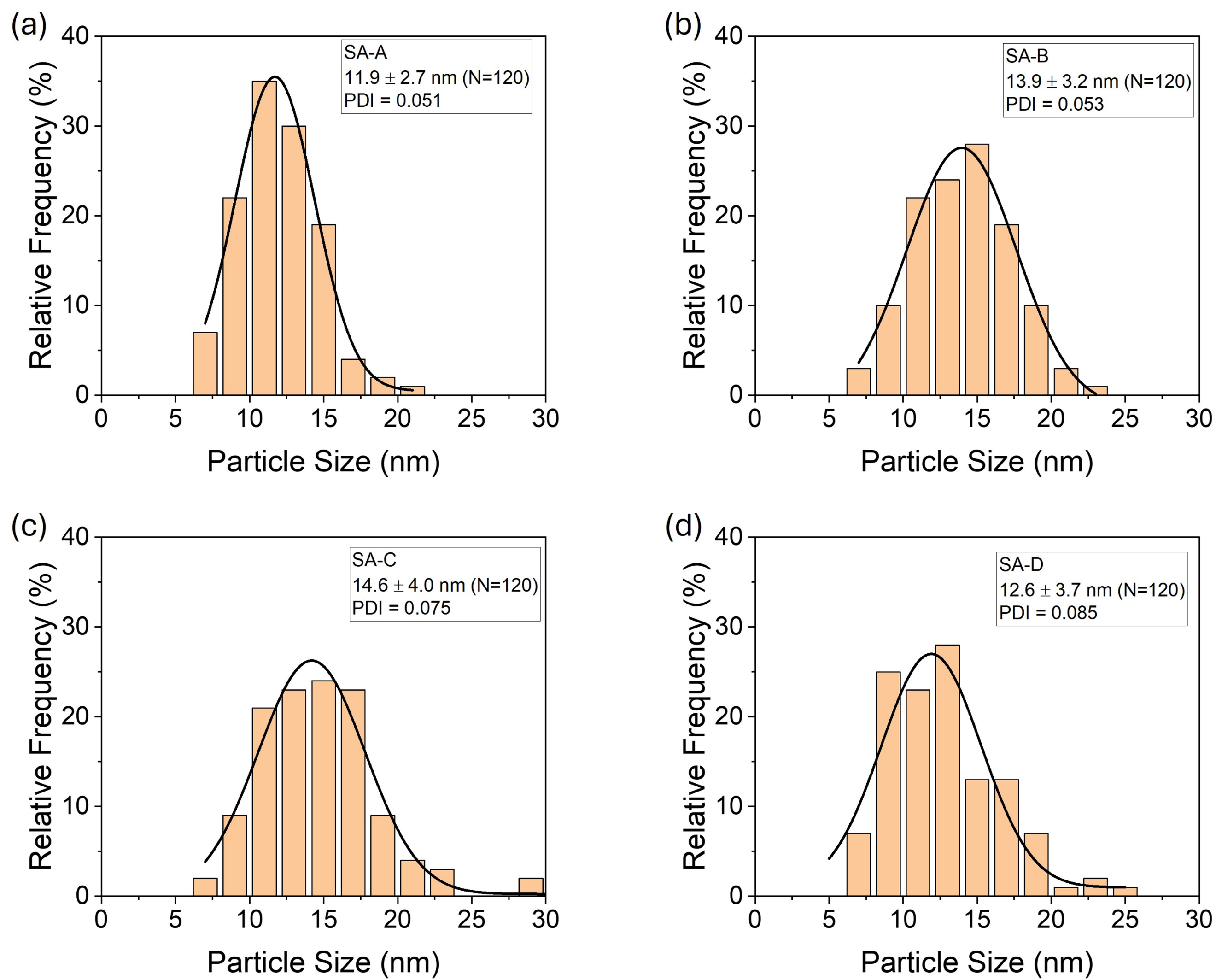


**Figure S1**. Size distribution histograms obtained by TEM micrographs of the SA-X set: **(a)** SA-A, **(b)** SA-B, **(c)** SA-C, and **(d)** SA-D. Size distribution was performed using ImageJ software.

**Table S1.** % of non-magnetic material (PEG or SiO_2_) and magnetic material (iron oxide) determined by TGA and ICP-OES, hydrodynamic size (D_H_), and polydispersity index (PDI) determined by DLS, as well as D_TEM_ obtained from TEM micrographs of the soft and dense aggregates.

| Sample | % Non-magnetic material | % Magnetic Material | D_H_ (nm) | PDI | D_TEM_ (nm) |
| --- | --- | --- | --- | --- | --- |
| SA-A  DA-A | 6.3  80.8 | 93.7  19.2 | 135.9  231.1 | 0.16  0.06 | 11.9  ≈ 200 |
| SA-B  DA-B | 2.3  82.8 | 97.7  17.2 | 140.0  388.0 | 0.09  0.05 | 13.9  ≈ 200 |
| SA-C  DA-C | 3.9  74.5 | 96.1  25.5 | 144.2  337.9 | 0.10  0.02 | 14.6  ≈ 200 |
| SA-D  DA-D | 8.87  80.1 | 91.1  19.9 | 195.8  352.5 | 0.08  0.02 | 12.6  ≈ 200 |


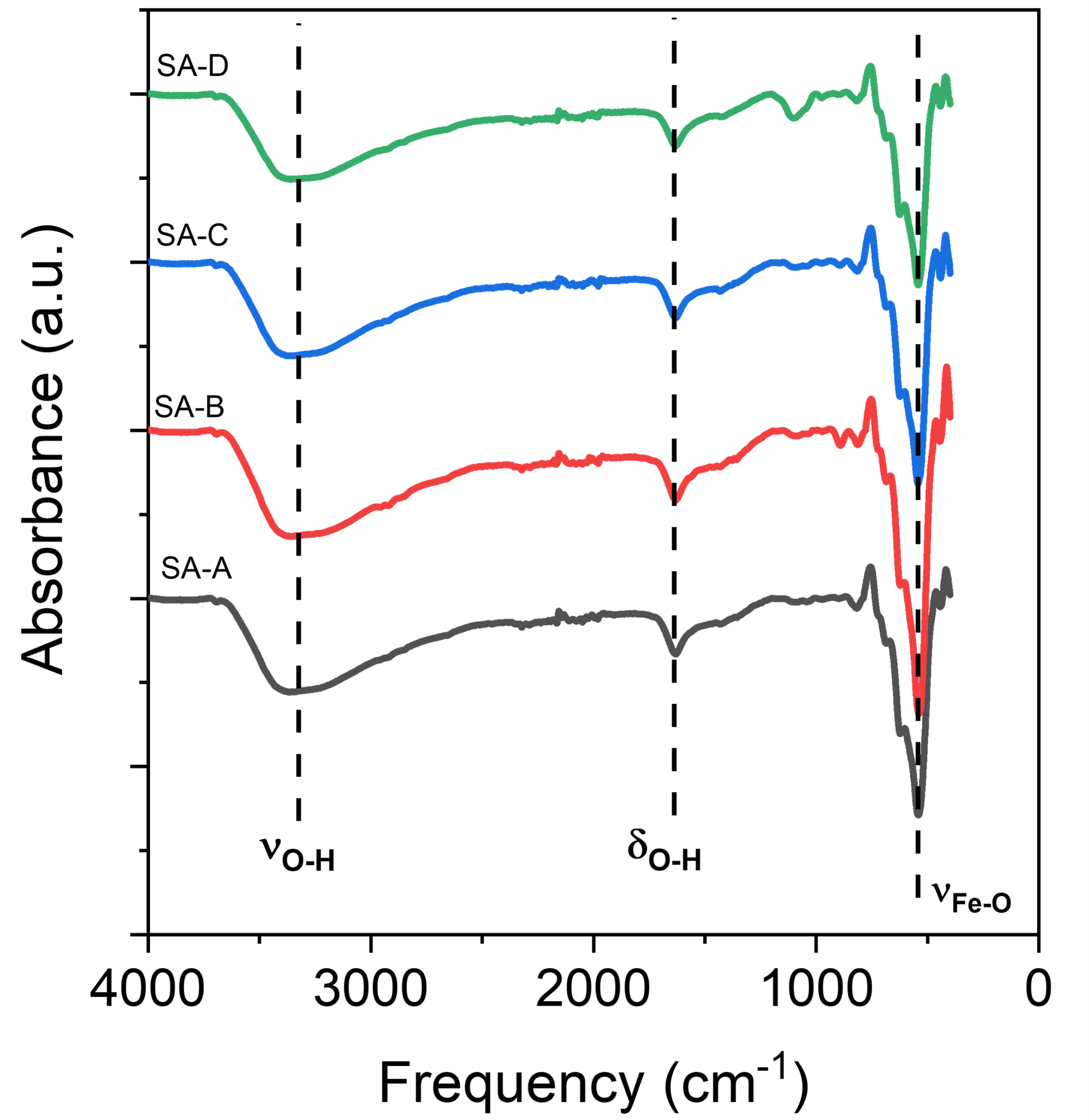


**Figure S2**. FTIR spectra of SA-X set of MNPs: SA-A (gray), SA-B (red), SA-C (blue) and SA-D (green).


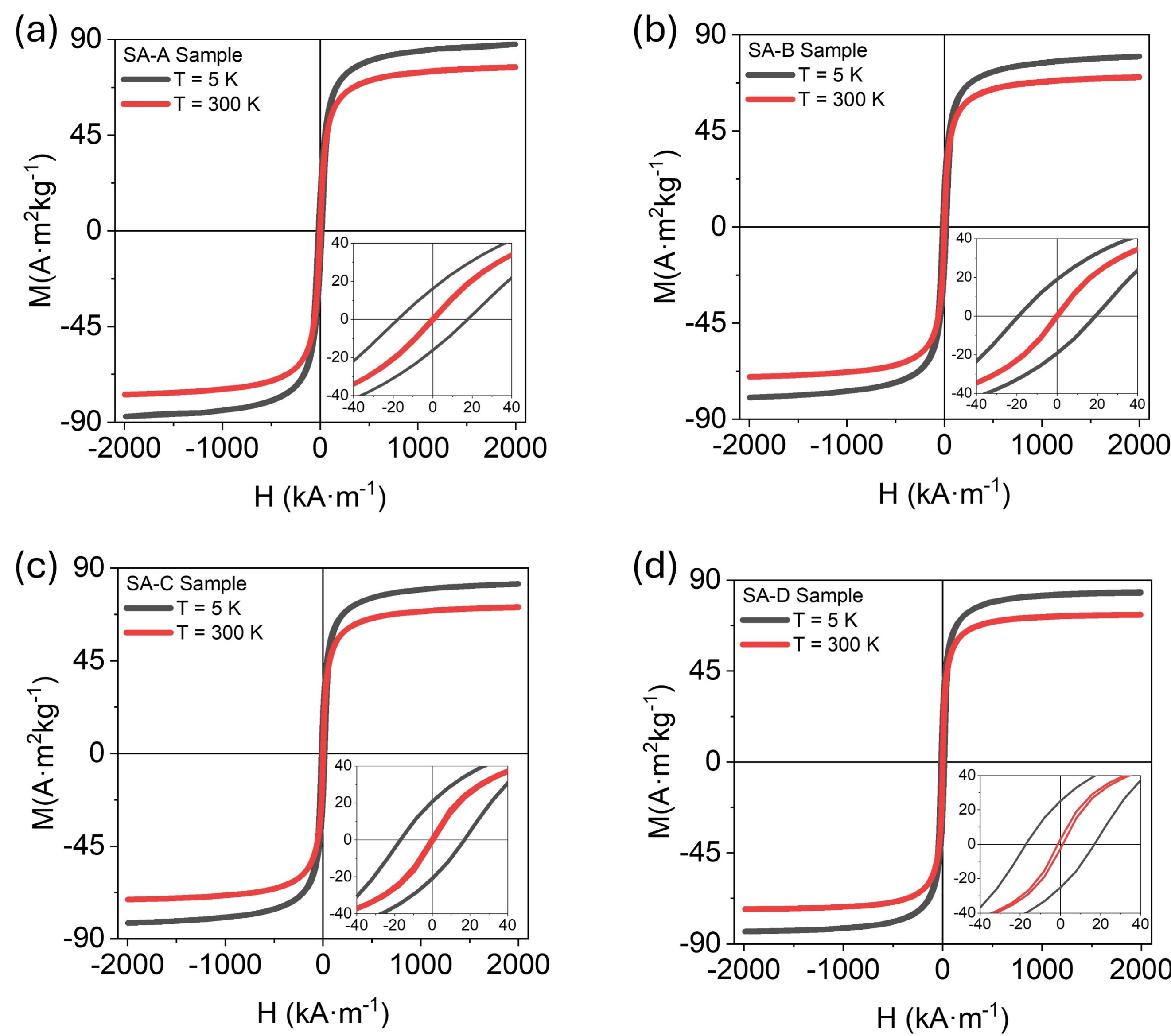


**Figure S3.** Hysteresis loops obtained at T = 5 K (gray line) and T = 300 K (red line) for the samples (a) SA-A, (b) SA-B, (c) SA-C, and (d) SA-D. Insets show the scale amplification of the hysteresis loops.


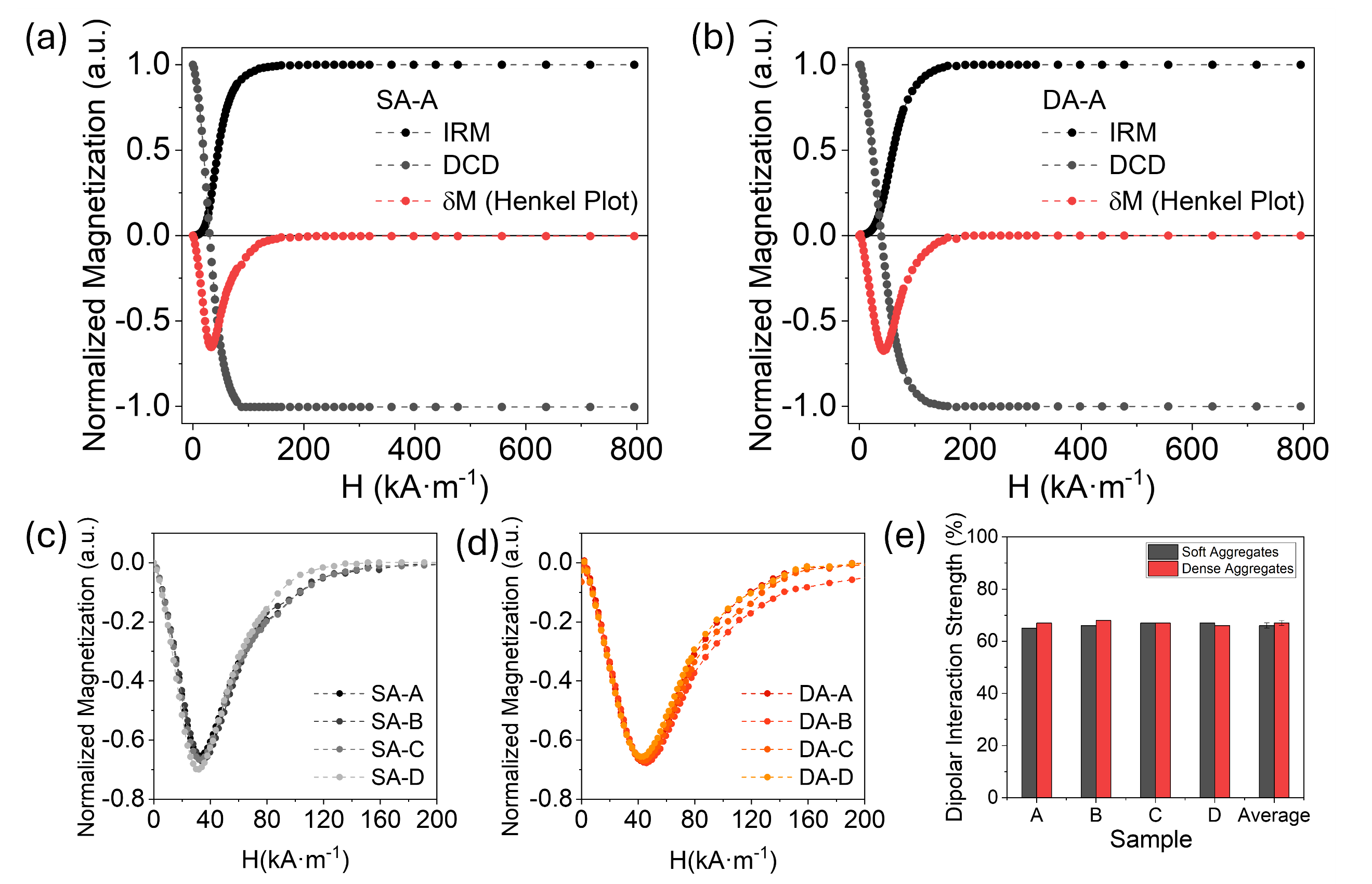


**Figure S4**. Representative normalized isothermal remanent magnetization (IRM), direct current demagnetization (DCD), and δM curves (Henkel plots) measured at 5 K for (a) soft aggregates (SA-A sample) and (b) dense aggregates (DA-A sample). δM curves (Henkel plots) for the entire batch of (c) soft aggregates and (d) dense aggregates. (e) Dipolar interaction strength (%) quantified from the maximum of the δM peak obtained via Henkel plot analysis for the soft (red bars) and dense aggregates (black bars).

**
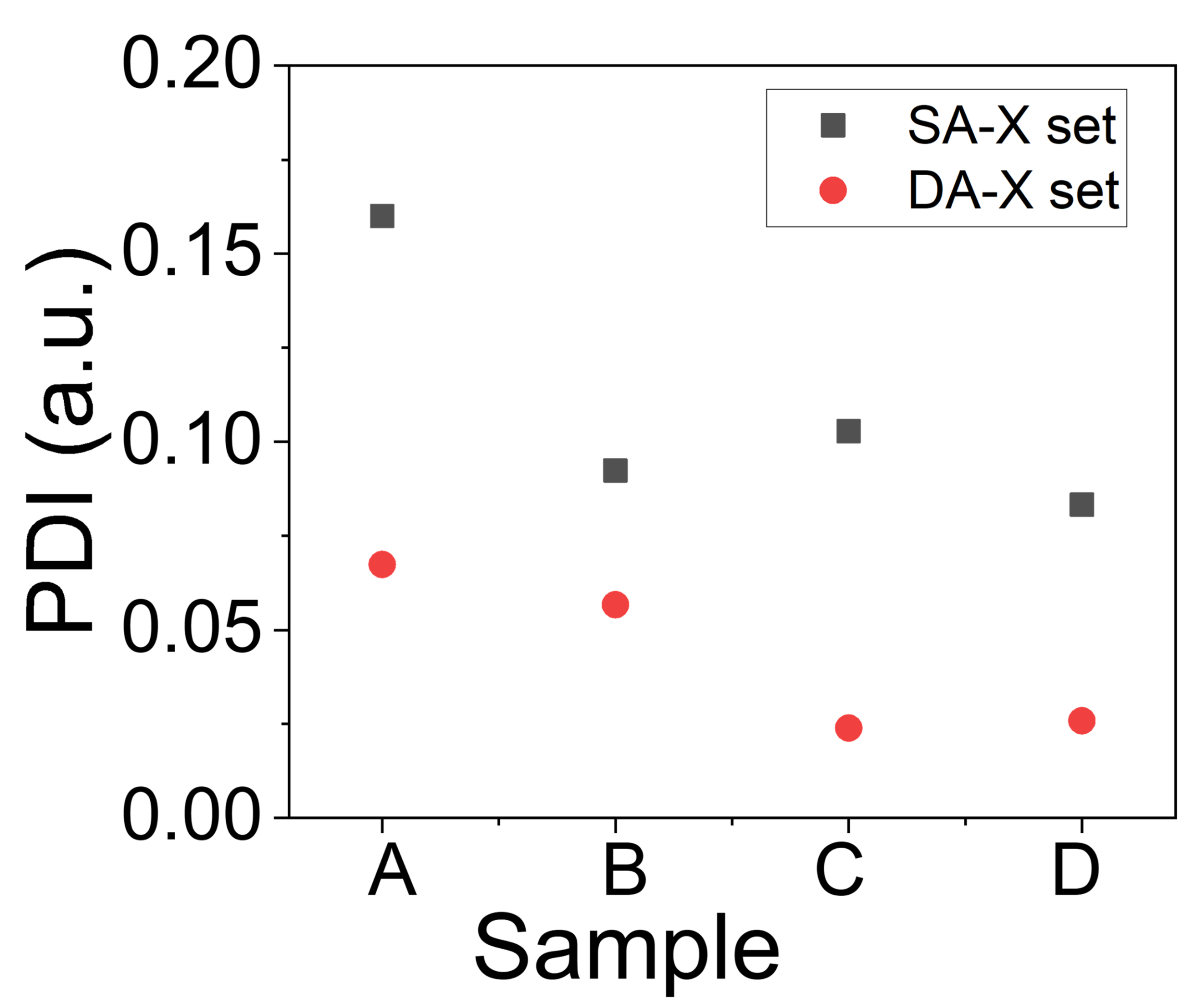
**

**Figure S5.** PDI obtained by DLS for the soft aggregates (gray points) and dense aggregates (red points).


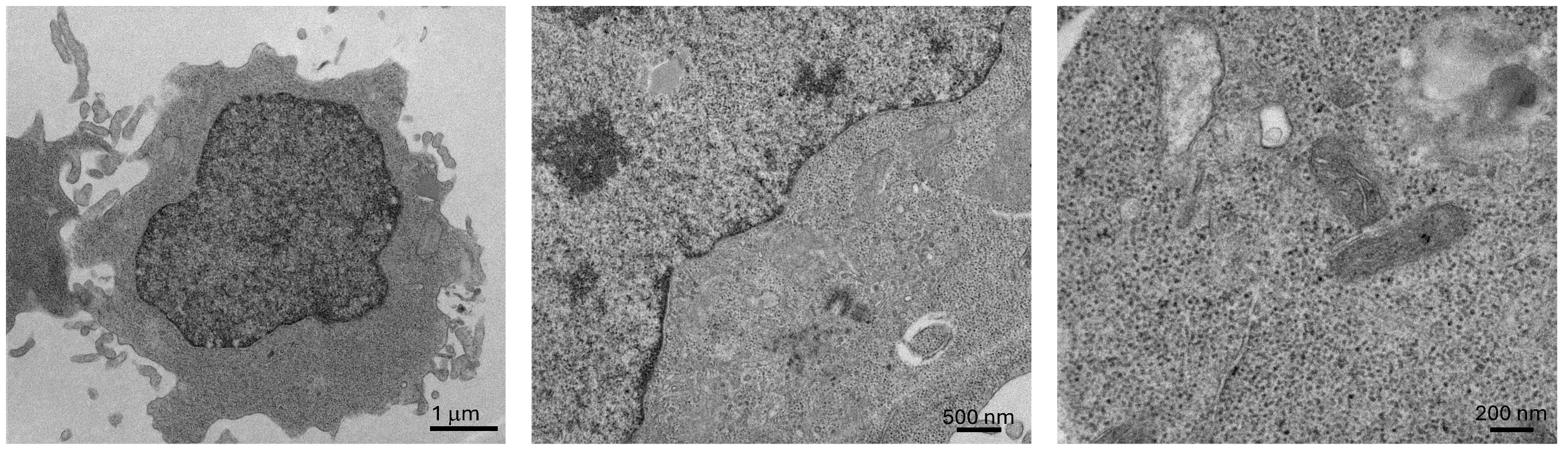


**Figure S6.** TEM images of ultrathin sections of untreated control T lymphocytes.


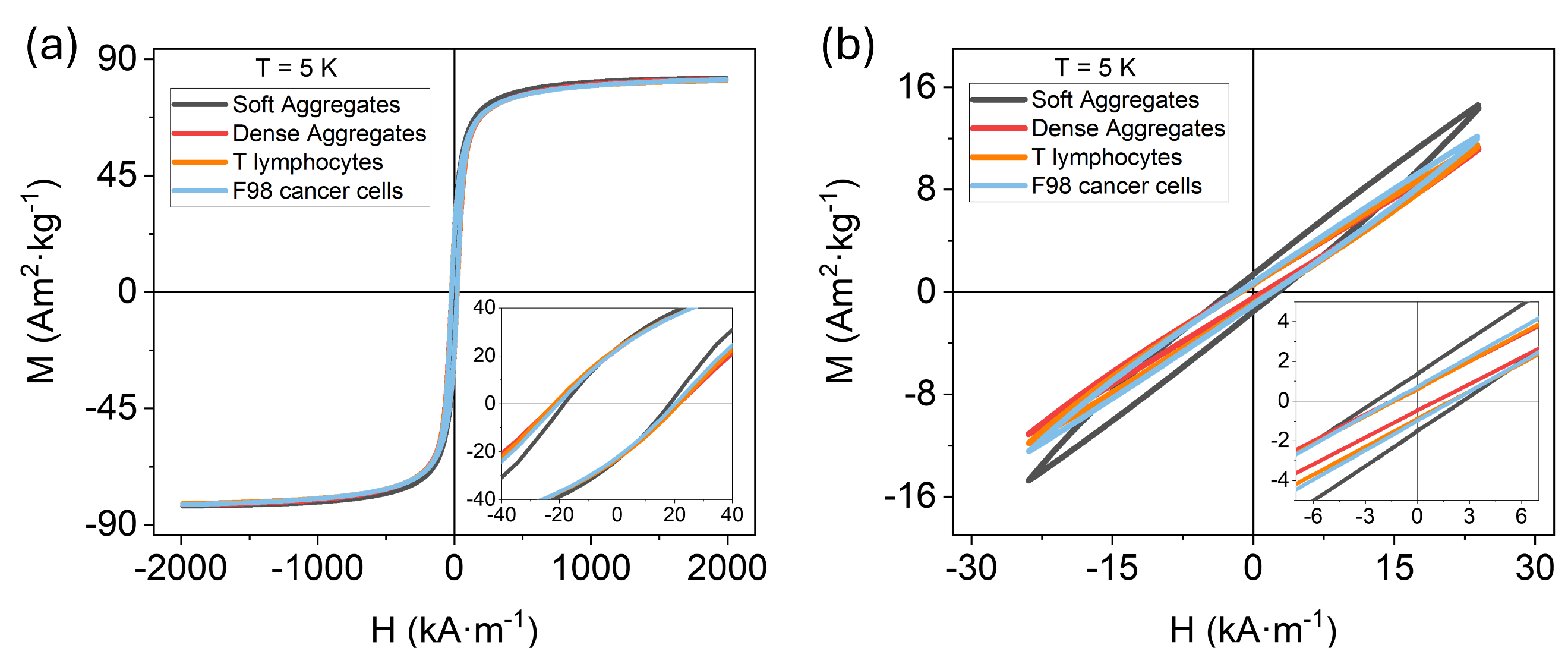


**Figure S7.** Hysteresis loops of chemically induced soft and dense aggregates and naturally occurring aggregates in T lymphocytes and F98 cancer cells, measured at T = 5 K under (a) high-field conditions (−2000 kA·m^−1^ to +2000 kA·m^−1^) and (b) low-field conditions (−24 kA·m^−1^ to +24 kA·m^−1^). Colors denote aggregation types: soft aggregates (gray), dense aggregates (red), naturally induced aggregation in T lymphocytes (orange), and in cancerous F98 cells (blue).


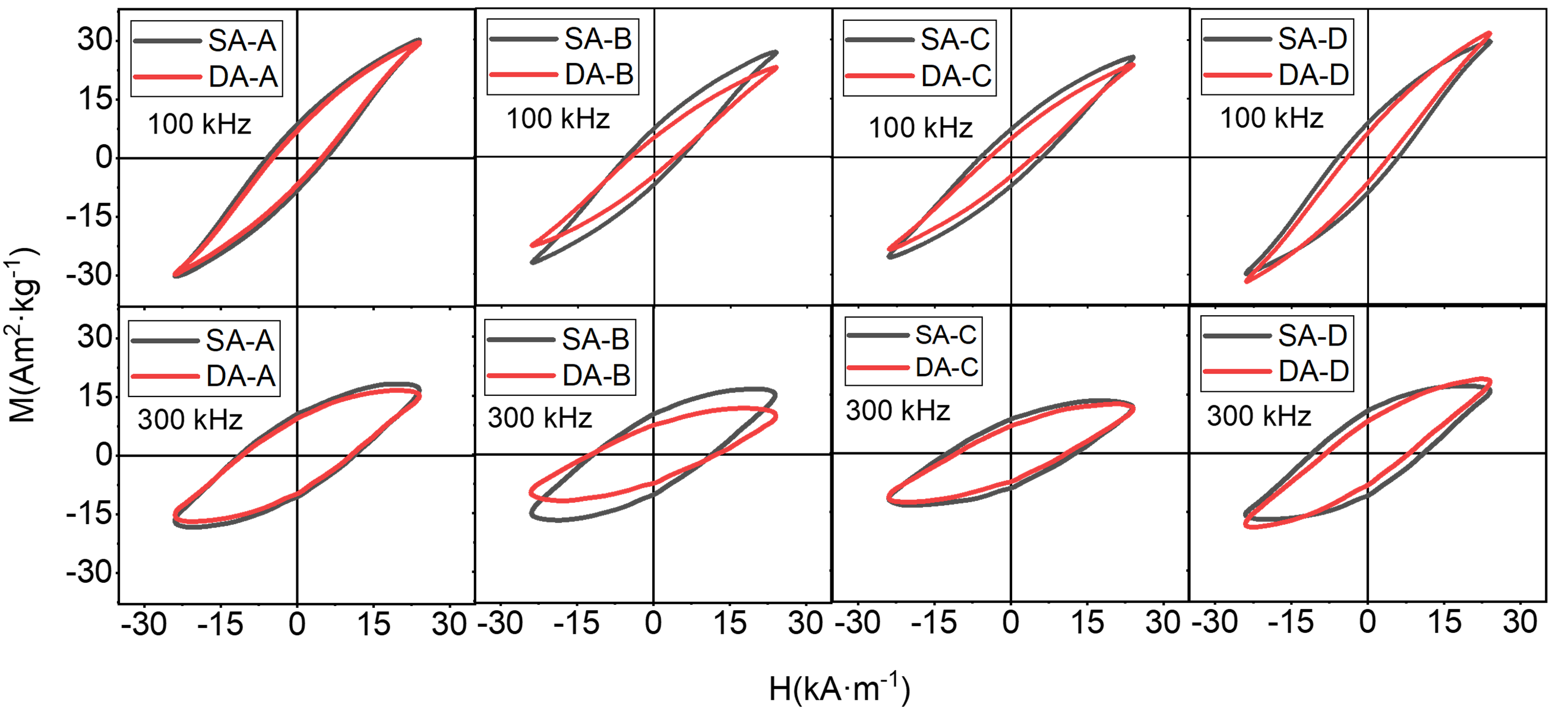


**Figure S8.** Hysteresis loops under AC conditions, 24 kA·m^−1^ and 100 kHz (top, a-d) and 24 kA·m^−1^ and 300 kHz (bottom, e-h) of the SA-X set (black curves) and the corresponding aggregated MNPs, DA-X (red curves). The MNPs were dispersed in a mixture of PEG:H_2_O 90:10 at [Fe_3_O_4_] = 0.50 mg·mL^-1^, with a viscosity of η = 83.70 mPa^·^s.


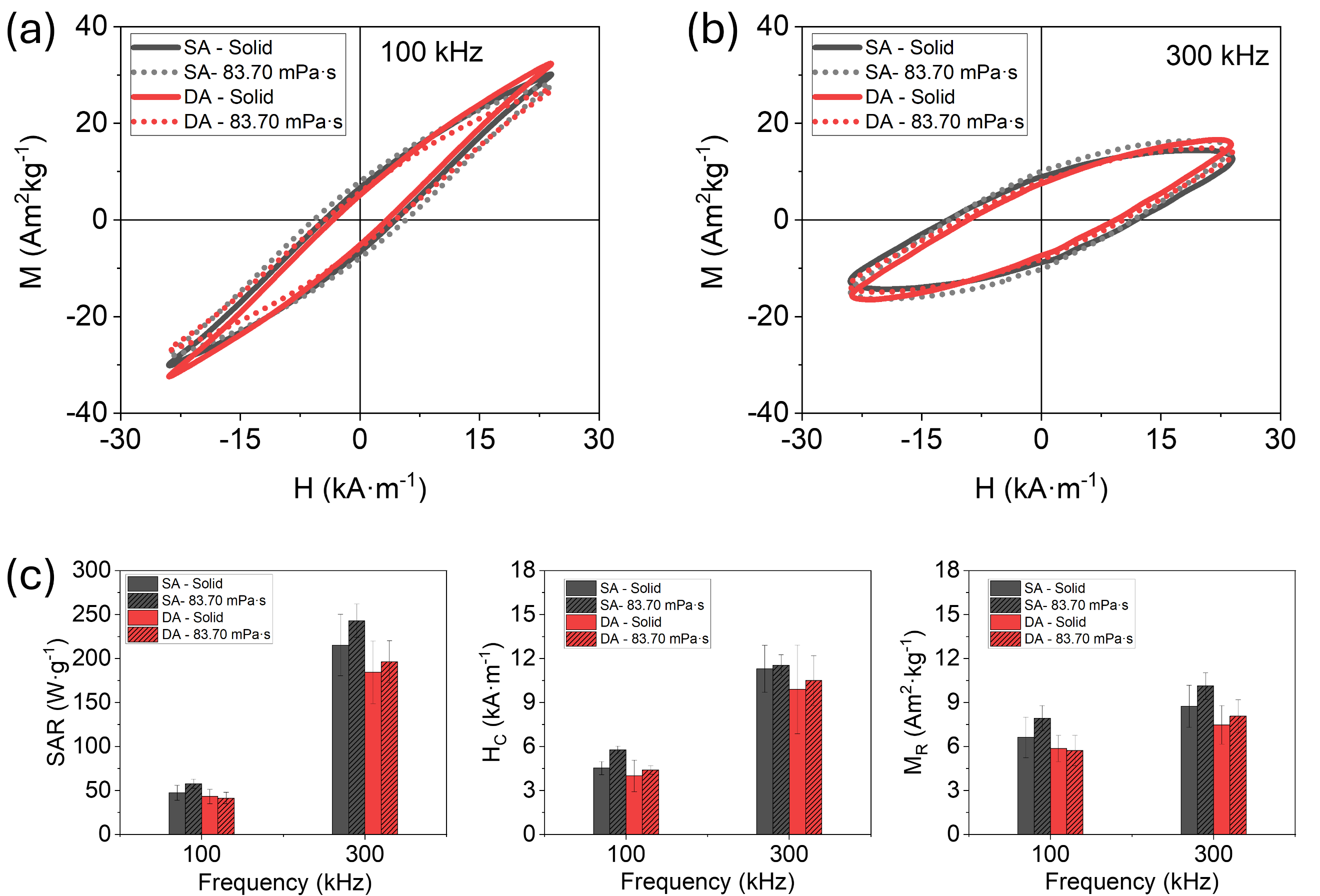


**Figure S9.** Hysteresis loops under alternating AC conditions for the SA-X (soft aggregates, black curves) and DA-X (dense aggregates, red curves) samples measured under two frequencies: (a) 24 kA·m^−1^ and 100 kHz, and (b) 24 kA·m^−1^ and 300 kHz. For each sample, measurements were performed under high-viscosity conditions (dotted lines) and in the solid state (solid lines). (c) Comparison of SAR, H_C_ and M_R_ for SA-X (black) and DA-X (red) samples measured under high-viscosity and solid-state conditions. Bars with fill patterns represent high-viscosity measurements, while solid-colored bars correspond to solid-state data.
